# Associations of dietary polyunsaturated fatty acids and sleep patterns with lung cancer in southwest China: A case-control study

**DOI:** 10.1371/journal.pone.0358576

**Published:** 2026-09-30

**Authors:** Xinwei Zuo, Qing Zhou, Lilong Pang, Gaoting Li, Feng Long, Zihao Xiang, Xin Gong, Manyi Xu, Ting Chen, Xuemei Lian

**Affiliations:** 1 Center for Lipid Research, Key Laboratory of Molecular Biology for Infectious Diseases (Ministry of Education), The Second Affiliated Hospital, Chongqing Medical University, Chongqing, China; 2 Chongqing Center for Disease Control and Prevention, Chongqing, China; 3 College of Public Health, Chongqing Medical University, Chongqing, China; 4 Chongqing Nanan District Center for Disease Control and Prevention, Chongqing, China; 5 Chongqing Tongnan District Center for Disease Control and Prevention, Chongqing, China; Mae Fah Luang University School of Anti Aging and Regenerative Medicine, THAILAND

## Abstract

**Background:**

Evidence on the associations of polyunsaturated fatty acid (PUFA) intake and sleep patterns with lung cancer remains inconsistent. We aimed to examine these associations in southwest China and to explore whether the association between sleep patterns and lung cancer differed according to dietary PUFA intake.

**Methods:**

We conducted a matched case-control study in Chongqing, southwest China, from August 22, 2023, to September 30, 2024. Each lung cancer case was matched to two controls by age group (<45, 45–65, or >65 years) and sex. Dietary PUFA intake was assessed using a validated semi-quantitative food frequency questionnaire (SQFFQ), and sleep patterns were evaluated using a composite healthy sleep score based on five sleep characteristics. Multivariable conditional logistic regression models were used to estimate odds ratios (ORs) and 95% confidence intervals (CIs).

**Results:**

A total of 322 lung cancer cases and 644 matched controls were included. In continuous analyses, each 1-g/day increase in omega-3 PUFA intake was associated with lower odds of lung cancer (OR = 0.82, 95% CI: 0.70–0.96). In tertile-based analyses, participants in the highest versus lowest intake tertiles had lower odds of lung cancer across all three PUFA types (omega-3 PUFAs: OR = 0.50, 95% CI: 0.32–0.78; omega-6 PUFAs: OR = 0.50, 95% CI: 0.31–0.80; total PUFAs: OR = 0.48, 95% CI: 0.30–0.79). Favorable sleep patterns were also associated with lower odds of lung cancer compared with unfavorable sleep patterns (OR = 0.59, 95% CI: 0.40–0.87). Although lower stratum-specific ORs were observed in the higher omega-3 PUFA intake stratum, the multiplicative interaction tests were not statistically significant.

**Conclusion:**

Higher dietary omega-3 PUFA intake and favorable sleep patterns were each associated with lower odds of lung cancer in this matched case-control study. Associations for omega-6 and total PUFA intake were observed in tertile-based analyses but were not consistent across modeling approaches. These observational findings require confirmation in prospective studies.

## Introduction

Lung cancer remains the most commonly diagnosed malignancy and the leading cause of cancer-related mortality worldwide, with approximately 2.5 million new cases and 1.8 million deaths reported in 2022 [1]. Projections estimate that by 2050, the global incidence could reach 3.8 million cases annually, resulting in approximately 3.2 million deaths [2]. In China, lung cancer accounted for roughly 1.0 million new cases and 0.7 million deaths in 2022, with incidence and mortality rates approximately 1.7 and 1.6 times higher than the global averages, respectively [3]. Identifying potential risk factors therefore remains a priority for lung cancer prevention and control.

Lung cancer risk factors include non-modifiable determinants such as age, sex, and family history, as well as modifiable factors including smoking, environmental exposure, diet, and sleep. Among these, smoking is the leading contributor to the global disease burden [4]. Nonetheless, accumulating evidence suggests that dietary factors may also be associated with lung cancer [5]. Notably, dietary polyunsaturated fatty acids (PUFAs), including omega-3, omega-6, and total PUFA intakes, have been investigated for their possible associations with lung cancer risk, although results are inconsistent. Some studies have reported inverse associations between PUFA intake and lung cancer, whereas others have reported null or positive associations, particularly for omega-6 and total PUFA intake [6–9]. To date, few studies have examined these associations in populations from southwest China.

Sleep characteristics, including sleep duration, chronotype, insomnia, snoring, and daytime sleepiness, have increasingly been examined as lifestyle factors potentially related to lung cancer risk [10–12]. A growing body of evidence suggests that unfavorable sleep behaviors may influence carcinogenesis by impairing immune function, hormonal regulation, and DNA repair processes [13–16]. However, many previous studies have focused on individual sleep components rather than overall sleep patterns. To address this limitation, a composite healthy sleep score incorporating multiple sleep characteristics has been applied across various health outcomes [17], providing a broader assessment of sleep health.

Although prior subgroup analyses have examined whether associations between sleep and cancer vary according to genetic susceptibility, age, or body mass index (BMI) [18,19], to our knowledge, potential variation in the sleep–lung cancer association across dietary PUFA intake levels has not been examined. Shared inflammatory and metabolic pathways involving dietary PUFA intake and sleep may be relevant to carcinogenesis [6] and provide a rationale for this exploratory assessment.

The primary objective of this study was to examine the associations of dietary PUFA intake and sleep patterns with lung cancer in a population from southwest China. As a secondary objective, we conducted exploratory stratified analyses and formally assessed multiplicative interactions between sleep patterns and dietary PUFA intake.

## Materials and methods

### Study design and participants

We conducted a hospital- and community-based matched case-control study in Chongqing, southwest China, between August 22, 2023, and September 30, 2024. Cases were patients with newly or recently diagnosed lung cancer recruited from two sources: the Department of Thoracic Surgery at the Third Affiliated Hospital of Chongqing Medical University between October 2023 and September 2024, and the Chongqing Tumor Registration System, through which community-based cases were identified in August 2023 and recruited by local primary healthcare institutions from randomly sampled communities in Nanan District and Tongnan District. Cases were eligible if they: (i) had a definite histopathological diagnosis of lung cancer (ICD-10 C33-C34); (ii) were aged ≥18 years; (iii) had a disease duration of ≤2 years; and (iv) had a local Chongqing household registration or living in Chongqing for ≥20 years. Patients with concurrent malignancies or those who were pregnant or lactating were excluded.

The control group was recruited in August 2023 and July 2024 from multiple communities in Nanan District and Tongnan District, Chongqing, which were randomly selected using the same sampling procedure as the community-based case group. Community residents were recruited by local primary care institutions. Eligible controls were required to have no cancer record in the Chongqing Tumor Registration System and to self-report no current or previous history of cancer. They were also required to meet the same residential criteria as cases, namely, to have local household registration in Chongqing or to have lived in Chongqing for ≥20 years. Individuals who were pregnant or lactating were excluded. Cases and controls were matched at a ratio of 1:2 by sex and age group (<45, 45–65, or >65 years).

Of 1,177 participants initially enrolled (355 cases, 822 controls), 27 were excluded for not meeting eligibility criteria (19 cases, 8 controls) and a further 33 (8 cases, 25 controls) were excluded due to missing data on key variables or implausible energy intakes (men: < 800 or >4,200 kcal/day; women: < 600 or >3,500 kcal/day) [20]. This left 328 cases and 789 controls eligible for matching. After 1:2 matching by age group and sex, the final analytic sample comprised 322 cases and 644 matched controls (Fig 1).

**Fig 1:**
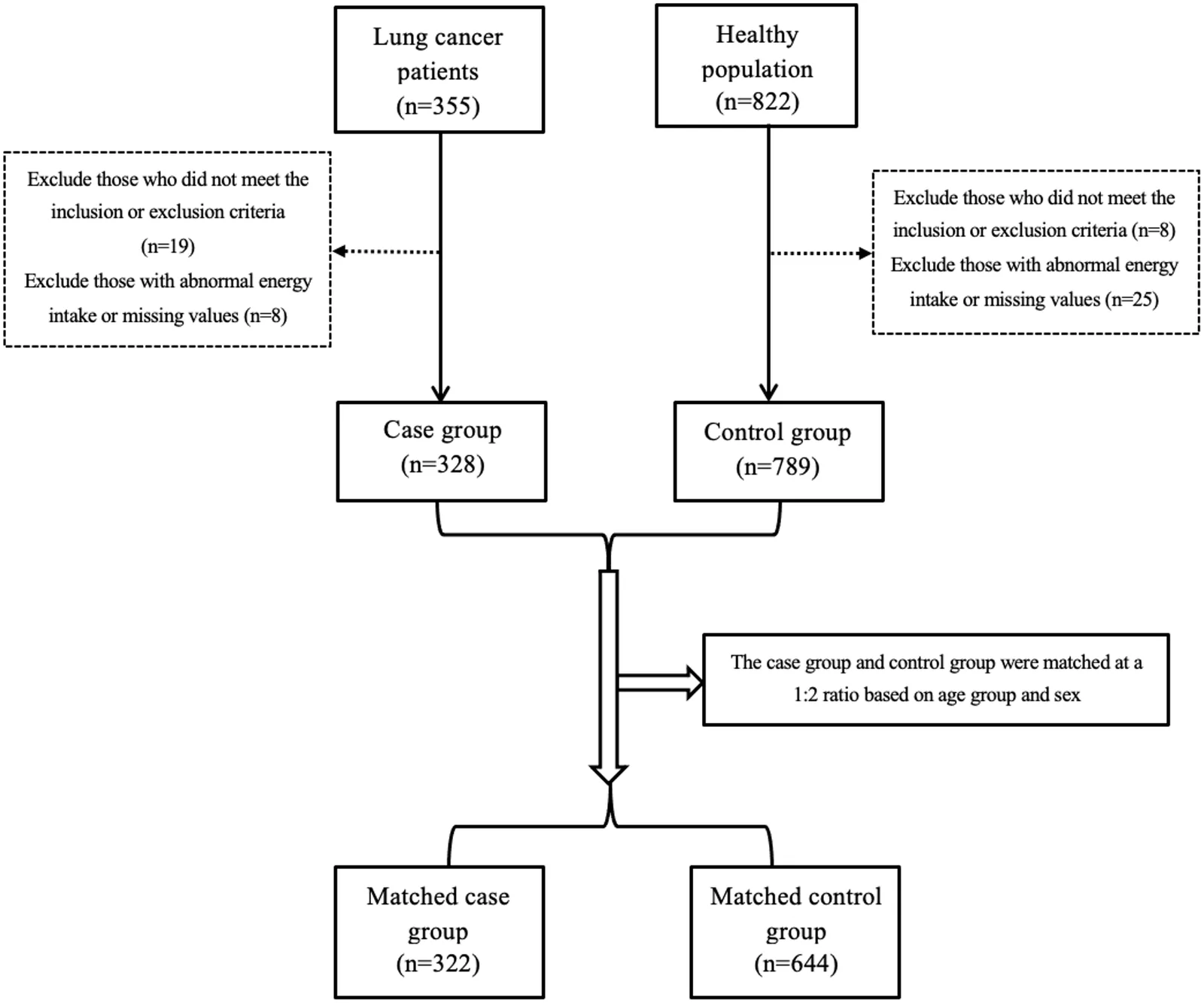
Flowchart of the analytic sample selection process.

*Sample size and selection.* The sample size was estimated based on previous epidemiological studies on dietary PUFAs, sleep quality, and lung cancer [6,21]. Using a 1:2 case-to-control matching ratio in PASS 11.0 (NCS, USA), the calculation indicated that at least 205 cases and 410 controls were required.

### Data collection and measurements

Prior to data collection, all participants provided written informed consent. Structured face-to-face interviews were conducted by trained and certified interviewers. All exposure information, including dietary intake and sleep behaviors, was collected retrospectively with reference to the 12 months preceding lung cancer diagnosis for cases, and the 12 months preceding questionnaire completion for controls.

*Assessment of dietary PUFAs*. Dietary intakes of omega-3, omega-6, and total PUFAs were assessed using a validated semi-quantitative food frequency questionnaire (SQFFQ) developed to reflect the dietary habits of urban and rural populations in Chongqing. The SQFFQ was originally developed from 3-day weighed food records from Chongqing adults and was designed to capture major food and nutrient intakes in this population. Its relative validity was evaluated in 92 healthy adults aged 35–55 years from urban and rural areas of Chongqing using 3-day weighed food records as the reference method, while its reproducibility was assessed in 48 adults of the same age range using repeated SQFFQ administrations at a 4-week interval [22].

Dietary intake was assessed over the 12 months preceding lung cancer diagnosis for cases and the 12 months preceding questionnaire completion for controls. The questionnaire included 119 food items grouped into 12 main food groups. Participants reported usual consumption frequency using eight predefined categories, ranging from 1–3 times per month to >3 times per day, and average portion size in grams for each food item. Trained interviewers used standardized household measures, including bowls, plates, and cups, together with a detailed food atlas, to assist participants in estimating portion sizes. The daily intake of each food item was calculated by converting the reported frequency to a daily rate and multiplying it by the reported portion size. Nutrient and energy intakes were estimated using the Chinese Food Composition Tables (Standard Edition, 6th Edition) [23]. Daily intakes of omega-3, omega-6, and total PUFA were calculated by summing the PUFA contributions from all reported food items and were expressed as grams per day.

*Assessment of sleep patterns and healthy sleep score.* Five sleep characteristics were assessed: sleep duration, chronotype, insomnia, snoring, and daytime sleepiness. *Sleep duration* was based on the question ‘Over the past year, excluding nap time, how many hours do you sleep on average each day?’; responses of 7–8 hours/day were considered favorable (1 point). *Chronotype* was assessed by asking, ‘How would you describe yourself over the past year? (i) definitely a “morning” person, (ii) more a “morning” than “evening” person, (iii) more an “evening” than “morning” person, or (iv) definitely an “evening” person over the past year’; morning-type responses (i or ii) were considered favorable (1 point). *Insomnia* was assessed by asking ‘Over the past year, have you had difficulty falling asleep or waking up in the middle of the night?’, with responses of (i) never/rarely, (ii) sometimes, or (iii) usually; never/rarely was considered favorable (1 point). *Snoring* was assessed by the question ‘Over the past year, have you snored? Or have your family or friends complained about your snoring?’, responses included (i) yes, or (ii) no; no snoring was considered favorable (1 point). *Daytime sleepiness* was captured by ‘Over the past year, how likely are you to doze off or fall asleep unintentionally during the day (e.g., while working, reading, or driving)?’, with responses of (i) never/rarely, (ii) sometimes, (iii) often, or (iv) all of the time; never/rarely was considered favorable (1 point).

A healthy sleep score (range, 0–5) was calculated by summing points across the five characteristics, with higher scores reflecting healthier overall sleep. Participants were classified into three categories based on the total score: unfavorable (0–1), medium (2–3), and favorable (4–5). This composite scoring approach has been previously applied to examine sleep patterns in relation to type 2 diabetes [24], heart failure [25], and arrhythmia [26].

### Statistical analyses

We summarized participant characteristics as mean (standard deviation [SD]) for normally distributed variables, median (interquartile range) for skewed variables, and number (percentage) for categorical variables. For comparisons between cases and controls a t‑test (for normally distributed variables) and a Mann–Whitney U test (for skewed variables) were used to analyze continuous variables, and a chi‑squared test was used to analyze categorical variables.

Multivariable conditional logistic regression was used to estimate ORs and 95% CIs. Three models were fitted, and all models were conditioned on the matched sets to account for the 1:2 matching by age group and sex. The crude model included no additional covariate adjustment. Based on previous studies [27–30], two multivariable models were fitted. Model 1 was adjusted for education level (primary education or illiterate, junior high school, high school, or junior college or above), yearly household income (CNY) (<60,000 or ≥60,000), family history of lung cancer (yes/no), smoking status (never smokers, current smokers, or former smokers), and fish oil supplementation habit (yes/no). Model 2 was further adjusted for diabetes at baseline (yes/no) and BMI (<24.0, 24.0–27.9 or ≥28.0 kg/m^2^), energy (kcal/day), alcohol intake frequency (frequent, daily to 3–4 times per week; occasional, 1–2 times per week to 1–3 times per month; rarely/never, less than once per month or rare/never), passive smoking (yes/no), occupational exposure (yes/no), and hot cooking oil exposure (low, none or minor; high, moderate or heavy).

The primary analyses examined the associations of dietary PUFA intake and sleep patterns separately with the odds of lung cancer. Dietary intakes of omega-3, omega-6, and total PUFAs were modeled as continuous variables, with ORs estimated per 1-g/day increase. PUFA intake was additionally categorized into tertiles, with the lowest tertile used as the reference. Tests for linear trend were conducted by assigning the median value of each tertile as a continuous variable in the conditional logistic regression model. Sleep patterns were categorized as unfavorable, medium, and favorable, with the unfavorable category as the reference.

Secondary exploratory analyses examined stratum-specific associations between sleep patterns and lung cancer according to dietary PUFA intake. Each PUFA measure was dichotomized at the median to define lower- and higher-intake groups. All stratified analyses were adjusted for covariates included in Model 2. For each PUFA measure, multiplicative interaction was assessed by adding two cross-product terms to Model 2: medium sleep pattern × higher PUFA intake and favorable sleep pattern × higher PUFA intake.

All analyses were performed using SPSS 25.0 and R 4.4.3. Two-sided *P* values <0.05 were considered statistically significant.

### Ethical approval

This study was approved by the Ethics Committee of Chongqing Center for Disease Control and Prevention (approval No.KY-2023-007-1 and No. KY-2023-007-2). All participants provided written informed consent before participation, and the study was conducted in accordance with the Declaration of Helsinki and relevant guidelines and regulations. Participants were informed of the study aims, foreseeable harms (e.g., psychological distress from sensitive questions), and potential societal benefits. Data confidentiality was protected through coded identifiers, removal of direct personal identifiers, and restricted access by authorized staff.

### Study registration

This observational matched case-control study was retrospectively registered in the Chinese Clinical Trial Registry (registration ID: ChiCTR2500106928) on July 31, 2025, after completion of participant recruitment and data collection, which took place from August 2023 to September 2024.

## Results

### Characteristics of the participants

Table 1 summarizes the characteristics of the 322 lung cancer cases and 644 matched controls. Compared with controls, cases were more likely to have a family history of lung cancer, higher annual household income, passive smoking, occupational exposure, and current or former smokers. Cases had a lower prevalence of diabetes at baseline and obesity (BMI ≥ 28 kg/m^2^). They were also more likely to have unfavorable sleep patterns and reported lower omega-3, omega-6, and total PUFA intakes than controls.

**Table 1:** Characteristics of participants according to lung cancer status.

Characteristics	Overall	Cases	Controls	*P* value
Number of participants	966	322	644	
Age, year, mean (SD)	64.43 (10.84)	64.39 (10.70)	64.45 (10.92)	0.935
Age, %				
< 45	54 (5.6)	18 (5.6)	36 (5.6)	1.000
45–65	384 (39.8)	128 (39.8)	256 (39.8)	
> 65	528 (54.7)	176 (54.7)	352 (54.7)	
Sex, %				
Male	525 (54.3)	175 (54.3)	350 (54.3)	1.000
Female	441 (45.7)	147 (45.7)	294 (45.7)	
Education level, %				
Primary education or illiterate	373 (38.6)	134 (41.6)	239 (37.1)	0.185
Junior high school	303 (31.4)	87 (27.0)	216 (33.5)	
High school	166 (17.2)	55 (17.1)	111 (17.2)	
Junior college or above	124 (12.8)	46 (14.3)	78 (12.1)	
Yearly household income (CNY),%				
< 60,000	656 (67.9)	204 (63.4)	452 (70.2)	0.032
≥ 60,000	310 (32.1)	118 (36.6)	192 (29.8)	
Family history of lung cancer, %				
No	878 (90.9)	271 (84.2)	607 (94.3)	<0.001
Yes	88 (9.1)	51 (15.8)	37 (5.7)	
Smoking status, %				
Never smokers	601 (62.2)	172 (53.4)	429 (66.6)	<0.001
Current smokers	225 (23.3)	82 (25.5)	143 (22.2)	
Former smokers	140 (14.5)	68 (21.1)	72 (11.2)	
Diabetes at baseline,%				
No	785 (81.3)	278 (86.3)	507 (78.7)	0.004
Yes	181 (18.7)	44 (13.7)	137 (21.3)	
BMI, kg/m^2^,%				
< 24	539 (55.8)	199 (61.8)	340 (52.8)	0.003
24-27.9	339 (35.1)	106 (32.9)	233 (36.2)	
≥ 28	88 (9.1)	17 (5.3)	71 (11.0)	
Fish oil supplementation habit,%				
No	945 (97.8)	319 (99.1)	626 (97.2)	0.061
Yes	21 (2.2)	3 (0.9)	18 (2.8)	
Energy, kcal, mean (SD)	1866.45 (627.45)	1811.21 (631.08)	1894.07 (624.29)	0.053
Alcohol intake frequency,%				
Frequent	183 (18.9)	69 (21.4)	114 (17.7)	0.110
Occasional	78 (8.1)	19 (5.9)	59 (9.2)	
Rare/never	705 (73)	234 (72.7)	471 (73.1)	
Passive smoking				
No	788 (81.6)	250 (77.6)	538 (83.5)	0.026
Yes	178 (18.4)	72 (22.4)	106 (16.5)	
Occupational exposure,%				
No	787 (81.5)	232 (72.0)	555 (86.2)	<0.001
Yes	179 (18.5)	90 (28)	89 (13.8)	
Hot cooking oil exposure,%				
Low	656 (67.9)	217 (67.4)	439 (68.2)	0.807
High	310 (32.1)	105 (32.6)	205 (31.8)	
Sleep patterns,%				
Unfavorable	240 (24.8)	93 (28.9)	147 (22.8)	0.035
Medium	355 (36.7)	122 (37.9)	233 (36.2)	
Favorable	371 (38.4)	107 (33.2)	264 (41.0)	
Omega-3 PUFA intake, g/d, mean (SD)	2.61 (1.24)	2.44 (1.23)	2.70 (1.24)	0.002
Omega-6 PUFA intake, g/d, mean (SD)	12.78 (6.31)	11.96 (6.54)	13.20 (6.16)	0.004
Total PUFA intake, g/d, mean (SD)	20.76 (9.52)	19.86 (9.97)	21.21 (9.26)	0.037

Abbreviations: BMI, body mass index, PUFA, polyunsaturated fatty acid. Data are presented as mean (SD) for normally distributed variables and number (percentage) for categorical variables. BMI was calculated as weight in kilograms divided by the square of the height in meters.

### Association between dietary PUFA intake and lung cancer

Table 2 shows the associations between dietary PUFA intake and lung cancer. When modeled continuously, higher omega-3 PUFA intake was consistently associated with lower odds of lung cancer across all models. In Model 2, each 1-g/day increase in omega-3 PUFA intake was associated with lower odds of lung cancer (OR = 0.82, 95% CI: 0.70–0.96; *P* = 0.012). Neither omega-6 PUFA intake nor total PUFA intake was statistically significantly associated with lung cancer in Model 2 (omega-6 PUFA intake: OR = 0.97, 95% CI: 0.94–1.00; *P* = 0.090; total PUFA intake: OR = 0.99, 95% CI: 0.97–1.01; *P* = 0.377).

**Table 2:** Association between dietary PUFA intake and lung cancer.

Variables	Crude model		Model 1		Model 2	
	OR (95%CI)	*P* value	OR (95%CI)	*P* value	OR (95%CI)	*P* value
**Omega-3 PUFA intake**						
Continuous	0.83[0.74,0.94]	0.002	0.84[0.74,0.96]	0.008	0.82[0.70,0.96]	0.012
Categorical						
Tertile 1	1 (reference)		1 (reference)		1 (reference)	
Tertile 2	0.92[0.67,1.26]	0.592	0.88[0.62,1.24]	0.470	0.88[0.61,1.29]	0.535
Tertile 3	0.57[0.41,0.80]	0.001	0.55[0.38,0.80]	0.002	0.50[0.32,0.78]	0.002
*P*_trend_		0.001		0.001		0.002
**Omega-6 PUFA intake**						
Continuous	0.97[0.95,0.99]	0.004	0.97[0.95,1.00]	0.027	0.97[0.94,1.00]	0.090
Categorical						
Tertile 1	1 (reference)		1 (reference)		1 (reference)	
Tertile 2	0.73[0.52,1.00]	0.055	0.69[0.48,0.98]	0.039	0.65[0.44,0.98]	0.038
Tertile 3	0.54[0.39,0.76]	<0.001	0.57[0.40,0.82]	0.002	0.50[0.31,0.80]	0.004
*P*_trend_		<0.001		0.003		0.006
**Total PUFA intake**						
Continuous	0.98[0.97,1.00]	0.036	0.99[0.97,1.00]	0.102	0.99[0.97,1.01]	0.377
Categorical						
Tertile 1	1 (reference)		1 (reference)		1 (reference)	
Tertile 2	0.90[0.65,1.25]	0.514	0.82[0.58,1.17]	0.275	0.82[0.55,1.22]	0.329
Tertile 3	0.55[0.39,0.78]	0.001	0.56[0.39,0.81]	0.002	0.48[0.30,0.79]	0.003
*P*_trend_		<0.001		0.002		0.003

Abbreviations: OR, odds ratio; CI: confidence Interval.

Crude model: no adjustment.

Model 1: adjusted for education level, yearly household income, family history of lung cancer, smoking status, and fish oil supplementation habit.

Model 2: additionally adjusted for diabetes at baseline, BMI, energy, alcohol intake frequency, passive smoking, occupational exposure and hot cooking oil exposure.

In the tertile-based analyses, participants in the highest versus lowest tertile had lower odds of lung cancer for all three PUFA measures in Model 2 (omega-3 PUFA intake: OR = 0.50, 95% CI: 0.32–0.78; *P* = 0.002; omega-6 PUFA intake: OR = 0.50, 95% CI: 0.31–0.80; *P* = 0.004; total PUFA intake: OR = 0.48, 95% CI: 0.30–0.79; *P* = 0.003). Similar associations were observed in the crude model and Model 1. Trends toward lower odds across tertiles were also observed for omega-3, omega-6, and total PUFA intake (*P* for trend = 0.002, 0.006, 0.003 for omega-3 PUFAs, omega-6 PUFAs, and total PUFAs, respectively).

### Association between sleep patterns and lung cancer

Table 3 presents the association between sleep patterns and lung cancer. The overall association between sleep pattern category and lung cancer odds was statistically significant in Model 2 (*P* = 0.031). Compared with participants with unfavorable sleep patterns, those with favorable sleep patterns had lower odds of lung cancer after full adjustment (OR = 0.59, 95% CI: 0.40–0.87; *P* = 0.008). Similar associations were observed in the crude model and Model 1. The medium sleep pattern category was not statistically significantly associated with lung cancer in any model.

**Table 3:** Association of sleep patterns and lung cancer.

Variables	Crude model		Model 1		Model 2	
	OR (95%CI)	*P* value	OR (95%CI)	*P* value	OR (95%CI)	*P* value
Sleep patterns		0.040		0.009		0.031
Unfavorable	1 (reference)		1 (reference)		1 (reference)	
Medium	0.84[0.60,1.18]	0.304	0.78[0.54,1.13]	0.186	0.74[0.50,1.08]	0.120
Favorable	0.65[0.46,0.91]	0.012	0.57[0.39,0.82]	0.002	0.59[0.40,0.87]	0.008

Sleep patterns were calculated from the scores of sleep duration, chronotype, insomnia, snoring, and daytime sleepiness.

Crude model: no adjustment.

Model 1: adjusted for education level, yearly household income, family history of lung cancer, smoking status, and fish oil supplementation habit.

Model 2: additionally adjusted for diabetes at baseline, BMI, energy, alcohol intake frequency, passive smoking, occupational exposure and hot cooking oil exposure.

Component-specific analyses for each of the five sleep characteristics, including sleep duration, chronotype, insomnia, snoring, and daytime sleepiness are presented in S1 Table. Compared with definite evening types, definite morning types, more morning than evening types, and more evening than morning types were associated with lower odds of lung cancer across all models. No overall associations with lung cancer odds were observed for other sleep characteristics.

### Associations of dietary PUFAs and sleep patterns with lung cancer

Associations between sleep patterns and lung cancer stratified by dietary PUFA intake levels are presented in Fig 2. All estimates were adjusted for the covariates included in Model 2. In exploratory analyses stratified by omega-3 PUFA intake, medium and favorable sleep patterns were associated with lower odds of lung cancer, relative to unfavorable sleep patterns, within the higher-intake stratum (medium: OR = 0.40, 95% CI: 0.17–0.94; *P* = 0.035; favorable: OR = 0.23, 95% CI: 0.08–0.62; *P* = 0.004). The corresponding associations in the lower-intake stratum were not statistically significant, and no statistically significant within-stratum associations were observed for omega-6 or total PUFA intake (all *P* >0.05).

**Fig 2:**
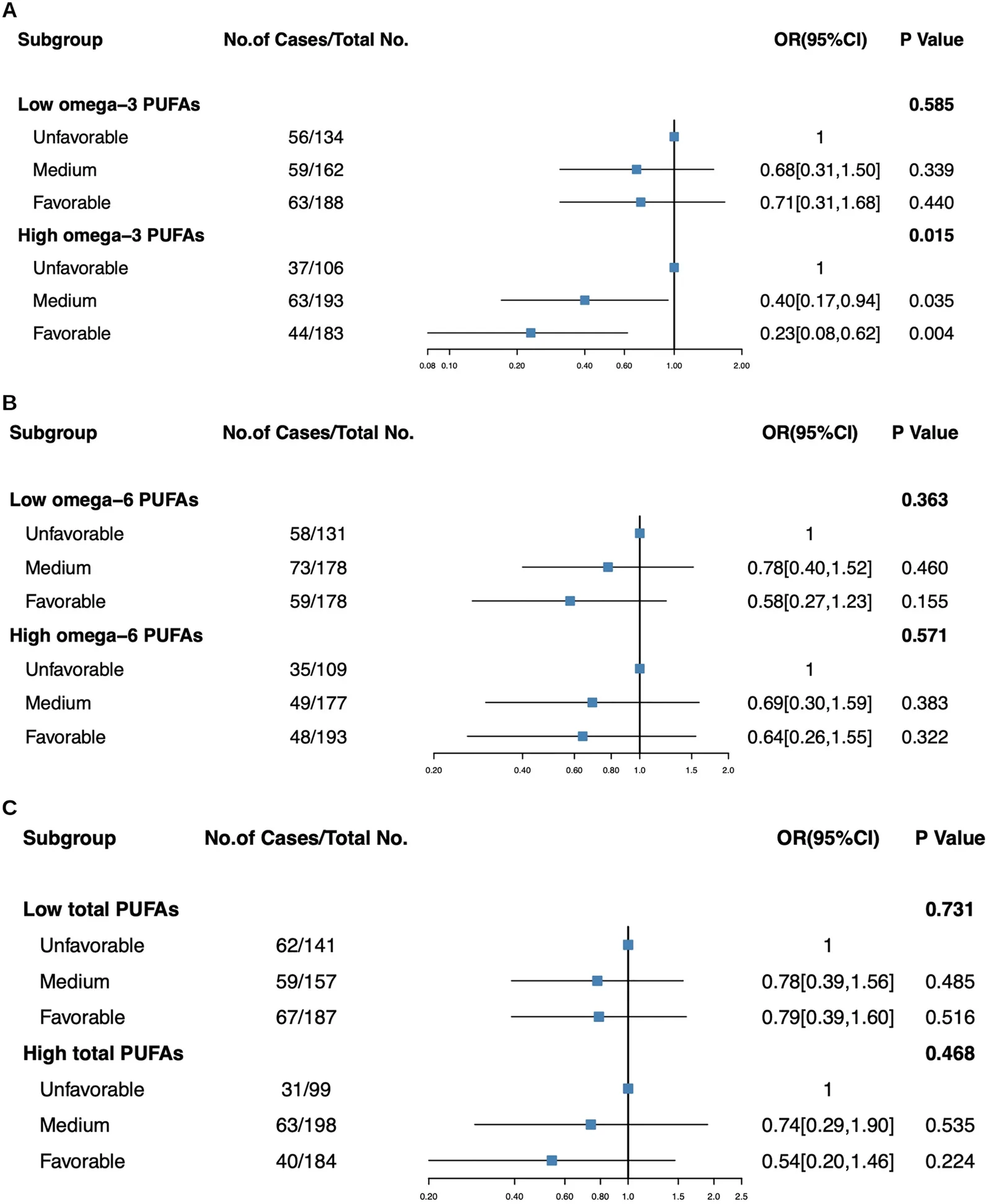
Associations between sleep patterns and lung cancer according to dietary PUFA intake levels. ORs and 95% CIs for medium and favorable sleep patterns, relative to unfavorable sleep patterns, are presented separately for lower and higher levels of (A) Omega-3 PUFA intake, (B) Omega-6 PUFA intake, and (C) Total PUFA intake. Participants were classified into low- and high-intake groups based on median intake values (omega-3 PUFAs: 2.43 g/day; omega-6 PUFAs: 11.41 g/day; total PUFAs: 18.47 g/day). Within each stratum, unfavorable sleep patterns served as the reference category. “No. of Cases/Total No.” indicates the number of lung cancer cases and the total number of participants in each sleep-pattern category within each stratum. Estimates were obtained from multivariable conditional logistic regression models adjusted for the covariates included in Model 2. All *P* values for the multiplicative interaction terms were >0.05.

For each PUFA measure, two cross-product terms were used to assess multiplicative interaction: medium sleep pattern × higher PUFA intake and favorable sleep pattern × higher PUFA intake, with unfavorable sleep pattern and lower PUFA intake as the reference categories. None of the interaction terms was statistically significant for omega-3 PUFA (*P* = 0.925 and 0.279), omega-6 PUFA (*P* = 0.391 and 0.703), or total PUFA (*P* = 0.648 and 0.394).

## Discussion

In this matched case-control study conducted in southwest China, higher omega-3 PUFA intake and favorable sleep patterns were each associated with lower odds of lung cancer. Associations with lower odds for omega-6 and total PUFA intake were observed mainly in tertile-based analyses and were not statistically significant when intake was modeled continuously in Model 2. Exploratory stratified analyses showed variation in the magnitude of the estimates across PUFA intake strata, but none of the multiplicative interaction terms was statistically significant.

Our results are generally consistent with previous studies suggesting associations between higher dietary PUFA intake and lower odds of lung cancer [31–36]. Omega-3 PUFAs, including eicosapentaenoic acid (EPA) and docosahexaenoic acid (DHA), have anti-inflammatory and immunomodulatory properties and may modulate pro-inflammatory signaling pathways, such as nuclear factor-kappa B (NF-κB), which may influence oxidative stress and inflammatory processes involved in tumor development [37,38]. Evidence from other chronic disease settings also supports the relevance of these pathways, with recent systematic reviews and meta-analyses reporting inverse associations of omega-3 intake with age-related macular degeneration and diabetic retinopathy [39,40]. However, these studies also highlight variation according to omega-3 species, exposure source, study design, and outcome type, as well as uncertainty regarding supplementation and long-term preventive effects. Accordingly, our findings should be interpreted as observational associations rather than evidence that omega-3 supplementation prevents lung cancer.

The tertile-based findings for omega-6 and total PUFA intake should be interpreted cautiously because the corresponding continuous associations were not statistically significant after full adjustment. One possible explanation for this discrepancy is a nonlinear dose-response relationship. The continuous models assume an approximately constant change in the log odds of lung cancer for each 1-g/day increment across the full intake distribution. If the association is concentrated above a certain intake level and subsequently plateaus, such a model may attenuate the overall per-unit association, whereas tertile-based comparisons may reveal contrasts between participants with relatively low and high intakes that are not apparent from a single linear per-unit estimate. Nevertheless, these findings only suggest possible nonlinear or threshold/plateau dynamics and should not be interpreted as evidence of a definitive threshold effect. Although some omega-6 PUFAs and their metabolites have been linked to pro-inflammatory processes, epidemiological evidence regarding omega-6 PUFA intake and lung cancer remains inconsistent [41,42]. Total PUFA intake represents a mixture of fatty acids with potentially divergent biological effects and may also reflect broader dietary patterns and correlated behaviors. Other factors, including residual confounding and differences in model specification, may also have contributed to the discrepant findings. These findings should therefore be interpreted in the broader dietary and behavioral context rather than as evidence of isolated PUFA-specific effects.

In line with growing epidemiological evidence, we also found that favorable sleep patterns were associated with lower odds of lung cancer compared with unfavorable sleep patterns. Previous studies have reported associations of individual adverse sleep components, including short or long sleep duration, insomnia, and sleep-disordered breathing, with higher lung cancer occurrence or mortality [10–16]. For example, snoring, often indicating nocturnal hypoxia, and excessive daytime sleepiness have been associated with higher lung cancer occurrence [14], and frequent insomnia symptoms have also been associated with lung cancer [43]. By incorporating a composite score based on sleep duration, chronotype, insomnia, snoring, and daytime sleepiness, our study examined multiple dimensions of sleep health rather than a single component. Proposed mechanisms linking sleep disturbance to carcinogenesis include circadian dysregulation, oxidative stress, and chronic low-grade inflammation [44,45]. However, previous findings have been inconsistent and may partly reflect differences in populations, sleep measurement approaches, or residual confounding.

We further explored whether the association between sleep patterns and lung cancer differed across dietary PUFA intake strata. Lower stratum-specific ORs for medium and favorable sleep patterns were observed among participants with higher omega-3 PUFA intake. One possible biological framework is that sleep disruption and omega-3 PUFAs involve overlapping inflammatory, metabolic, circadian, oxidative stress, and immune pathways relevant to carcinogenesis. Experimental evidence suggests that sleep deficiency may promote lung tumorigenesis through CLOCK-regulated fatty acid metabolism, including ACSL1 activation, palmitoyl-CoA accumulation, and subsequent CLOCK stabilization [46]. Omega-3 PUFAs, particularly EPA and DHA, have anti-inflammatory and immunomodulatory properties, while DHA may also contribute to circadian and melatonin regulation through its role in pineal phospholipid composition [47]. Sleep deprivation may further impair immune regulation [48]. Evidence from other chronic disease settings also illustrates the broader relevance of mitochondrial dysfunction, oxidative stress, and lipid-related metabolic pathways to inflammation and tissue injury [49,50]. Omega-6 PUFAs comprise biologically diverse fatty acids and lipid mediators, whereas total PUFA intake combines fatty acids with potentially different effects; these broader measures may therefore correspond less specifically to pathways commonly attributed to omega-3 PUFAs [51,52]. Nevertheless, direct evidence linking dietary PUFA intake, sleep patterns, and lung cancer remains limited. Given the non-significant multiplicative interaction tests, median-based dichotomization of PUFA intake, and multiple comparisons across models and subgroups, these stratified findings should be considered hypothesis-generating rather than evidence of effect modification.

Several limitations of this study should be acknowledged. First, the case-control design precludes causal inference. Dietary intake and sleep behaviors were assessed retrospectively. Prediagnostic symptoms may have affected diet and sleep before diagnosis, while diagnosis or treatment may have influenced retrospective reporting, raising the possibility of recall bias and reverse causation. Second, residual confounding remains possible. Although smoking status (never, current, or former smokers) and passive smoking were included in the multivariable models, detailed cumulative smoking exposure data, including pack-years, smoking duration, cigarettes smoked per day, and smoking cessation history, were unavailable. Given the predominant role of smoking in lung cancer, residual confounding by smoking intensity and cumulative exposure therefore cannot be excluded. Residual confounding may also remain from overall diet quality, broader socioeconomic circumstances, cooking practices, health-conscious behaviors, night-shift work, and specific dietary factors such as red and processed meat intake. The lower prevalence of diabetes and obesity among cases than controls also warrants cautious interpretation and may reflect selection effects or residual confounding. Third, the median-based dichotomization may have reduced information and increased the instability of stratum-specific estimates. Fourth, the study was conducted in a specific population in southwest China, which may limit generalizability. Nevertheless, this study is among the first to investigate dietary PUFA intake and composite sleep patterns both independently and simultaneously in relation to lung cancer. The use of a multidimensional sleep score also enabled a broader assessment of sleep health than single-component measures.

## Conclusion

In summary, higher dietary omega-3 PUFA intake and favorable sleep patterns were each associated with lower odds of lung cancer in this case–control study. Associations with lower odds for omega-6 and total PUFA intake were observed in tertile-based analyses but were not consistently supported by continuous models. Exploratory stratified analyses provided no statistical evidence that dietary PUFA intake modified the association between sleep patterns and lung cancer. Further large-scale prospective studies in diverse populations are warranted to confirm these observational findings and clarify the relationships among PUFA intake, sleep patterns, and lung cancer.

## Supporting information

S1 Table Associations of sleep characteristics and lung cancer. (DOCX)

S1 File Data. (XLSX)

S2 File Analytic code. (SPS)

S3 File Code used to generate Fig 2. (R)
