# Supplementary material for: Associations of dietary polyunsaturated fatty acids and sleep patterns with lung cancer in southwest China: A case-control study
Source: PLoS One. 2026 Sep 30;21(9):e0358576. doi: 10.1371/journal.pone.0358576 (PMC13626354; doi:10.1371/journal.pone.0358576)
Supplement: Supplementary file 1 [file pone.0358576.s005.pdf]

Reviewer Report

Recommendation: Major Revision

General Comments

This is a relevant study examining dietary PUFAs, sleep patterns, and lung cancer risk in a case–control design. The sample size and overall approach are acceptable. However, the manuscript in its current form lacks conceptual clarity and tends to overinterpret the findings, particularly regarding interaction effects.

The combination of diet, sleep, and cancer is potentially interesting, but the biological rationale—especially from an inflammation perspective—is not sufficiently developed, making the study feel somewhat unfocused.

Major Comment

1. Conceptual clarity and rationale

   The manuscript attempts to link PUFAs, sleep, and lung cancer, but the underlying biological framework is weak. The discussion of inflammation and related mechanisms is superficial. A clearer rationale explaining how PUFA intake may interact with sleep-related pathways in carcinogenesis is needed.

2. Overinterpretation of interaction/stratified results

   Although stratified analyses are presented, the interaction tests are not statistically significant. Despite this, the manuscript emphasizes differential associations across PUFA strata. This is misleading. These findings should be clearly presented as exploratory, and the conclusions should be toned down.

3. Framing of objectives
   The study objectives are not clearly structured. The authors should distinguish between:

   - primary analyses (independent associations), and
   - secondary/exploratory analyses (stratification)

4. Confounding (especially smoking)
   Smoking is only adjusted as a categorical variable. Given its importance in lung cancer, more detailed adjustment (if available) or stratified analyses are recommended. This limitation should also be discussed more explicitly.
5. Statistical issues

   - Clarify whether conditional logistic regression was used given the matching design.
   - Dichotomization of PUFA intake (median split) should be justified or supplemented with more informative analyses (e.g., trends).

6. Strength of conclusions
   The conclusions are too strong for a case–control study. Statements implying prevention should be revised to reflect observational associations only.

Minor comments

- Language and grammar need editing.
- Some repetition between sections.
- Methods (sleep score, exposure timing) need clearer description.

Final Recommendation-Major Revision

The study is potentially publishable, but substantial revision is required to improve conceptual coherence and align interpretation with the data.
