# Supplementary material for: Associations of dietary polyunsaturated fatty acids and sleep patterns with lung cancer in southwest China: A case-control study
Source: PLoS One. 2026 Sep 30;21(9):e0358576. doi: 10.1371/journal.pone.0358576 (PMC13626354; doi:10.1371/journal.pone.0358576)
Supplement: Supplementary file 2 [file pone.0358576.s007.pdf]

General Comments

The authors have adequately revised the manuscript in response to the previous comments. The revised version is clearer and better organized than the original submission. The rationale linking dietary PUFAs, sleep patterns, and lung cancer has been strengthened, the objectives are now more clearly stated, and the statistical methods have been described more appropriately. The interpretation of the stratified analyses has also been moderated, with appropriate acknowledgement that the interaction tests were not statistically significant.

Overall, the manuscript has improved and is now suitable for publication after addressing a few minor issues.

Minor Comments

1. The authors have acknowledged the limitation regarding smoking adjustment in the Discussion. However, considering the major role of smoking in lung cancer, this limitation should be emphasized slightly more. If detailed smoking information (e.g., pack-years) is available, its inclusion would strengthen the analysis. If not, this should be clearly stated as a limitation.

2. For omega-6 and total PUFAs, the continuous analyses were not statistically significant after full adjustment, whereas the tertile analyses remained significant. A brief explanation for this difference (such as possible non-linear associations or threshold effects) would improve the discussion.

3. The reported registration date appears to be later than the study period. The authors should clarify whether the study was registered retrospectively.

4. The overall language has improved. However, a final proofreading would help to correct a few minor grammatical errors and improve readability.

Final Recommendation

The major concerns raised in the previous review have been satisfactorily addressed. The remaining issues are minor and can be resolved through revision of the manuscript. I recommend **Minor Revision**.
