# Supplementary material for: Associations of dietary polyunsaturated fatty acids and sleep patterns with lung cancer in southwest China: A case-control study
Source: PLoS One. 2026 Sep 30;21(9):e0358576. doi: 10.1371/journal.pone.0358576 (PMC13626354; doi:10.1371/journal.pone.0358576)
Supplement: Supplementary file 3 [file pone.0358576.s001.docx]

| **S1 Table.** Associations of sleep characteristics and lung cancer | | | | | | |
| --- | --- | --- | --- | --- | --- | --- |
| Variables | Crude model | | Model 1 | | Model 2 | |
|  | OR (95%CI) | *P* value | OR (95%CI) | *P* value | OR (95%CI) | *P* value |
| **Sleep duration** |  | 0.247 |  | 0.151 |  | 0.200 |
| <7 hours | 1 (reference) |  | 1 (reference) |  | 1 (reference) |  |
| 7–8 hours | 0.78[0.58,1.05] | 0.099 | 0.73[0.53,1.00] | 0.053 | 0.73[0.52,1.03] | 0.075 |
| >8 hours | 0.92[0.62,1.35] | 0.652 | 0.87[0.57,1.32] | 0.500 | 0.88[0.56,1.39] | 0.586 |
| **Chronotype** |  | <0.001 |  | <0.001 |  | <0.001 |
| Definite evening types | 1 (reference) |  | 1 (reference) |  | 1 (reference) |  |
| Definite morning types | 0.26[0.15,0.46] | <0.001 | 0.27[0.15,0.51] | <0.001 | 0.25[0.13,0.48] | <0.001 |
| More morning than evening types | 0.25[0.14,0.45] | <0.001 | 0.27[0.14,0.51] | <0.001 | 0.26[0.13,0.51] | <0.001 |
| More evening than morning types | 0.39[0.21,0.70] | 0.002 | 0.39[0.20,0.74] | 0.004 | 0.40[0.21,0.79] | 0.009 |
| **Insomnia** |  | 0.038 |  | 0.044 |  | 0.122 |
| Usually | 1 (reference) |  | 1 (reference) |  | 1 (reference) |  |
| Sometimes | 0.65[0.47,0.91] | 0.011 | 0.65[0.46,0.93] | 0.018 | 0.68[0.47,0.99] | 0.045 |
| Never/rarely | 0.74[0.53,1.04] | 0.084 | 0.70[0.49,1.00] | 0.049 | 0.75[0.51,1.10] | 0.135 |
| **Snoring** |  |  |  |  |  |  |
| Yes | 1 (reference) |  | 1 (reference) |  | 1 (reference) |  |
| No | 0.76[0.57,1.00] | 0.051 | 0.83[0.62,1.11] | 0.202 | 0.75[0.55,1.03] | 0.071 |
| **Daytime sleepiness** |  | 0.557 |  | 0.332 |  | 0.386 |
| All of the time | 1 (reference) |  | 1 (reference) |  | 1 (reference) |  |
| Never/rarely | 0.62[0.14,2.80] | 0.535 | 0.49[0.10,2.35] | 0.369 | 0.47[0.08,2.74] | 0.402 |
| Sometimes | 0.61[0.13,2.80] | 0.519 | 0.48[0.10,2.36] | 0.365 | 0.45[0.08,2.65] | 0.375 |
| Often | 0.86[0.18,4.02] | 0.847 | 0.77[0.15,3.85] | 0.745 | 0.72[0.12,4.39] | 0.724 |
| Crude model: no adjustment.  Model 1: adjusted for education level, family income, lung cancer family history, smoking status, and fish oil supplement habit.  Model 2: additionally adjusted for diabetes at baseline, BMI, energy, alcohol intake frequency, passive smoking, occupational exposure and hot cooking oil exposure. | | | | | | |
