# Supplementary material for: Associations of dietary polyunsaturated fatty acids and sleep patterns with lung cancer in southwest China: A case-control study
Source: PLoS One. 2026 Sep 30;21(9):e0358576. doi: 10.1371/journal.pone.0358576 (PMC13626354; doi:10.1371/journal.pone.0358576)
Supplement: Supplementary file 4 [file pone.0358576.s008.docx]

**Associations of dietary polyunsaturated fatty acids and sleep patterns with lung cancer in southwest China: a case-control study**

(Manuscript ID:PONE-D-26-10673)

Dear reviewers,

Thank you for your valuable comments. We have modified the manuscript, and revisions were highlighted in red to make sure changes could be easily visible to you. Please see our responses to the comments.

**Response to Reviewer 1 Comments**

***Point 1:*** *The manuscript contains obvious reference-management and manuscript-preparation errors that are not acceptable in its current form. The Introduction includes visible placeholders such as “Error! Reference source not found.” in the epidemiologic background and again in the sleep-related literature section, and a similar citation error appears for the Chinese Food Composition Table in the Methods. These errors are not trivial because they interrupt the scientific argument, obscure the evidentiary basis for key statements, and create concern regarding the overall care taken in manuscript preparation. These issues should be corrected throughout the full text, tables, figures, and supplementary materials before any further consideration.*

**Response 1:** We thank the reviewer for pointing out these important manuscript-preparation errors. We have carefully checked the full manuscript, including the Introduction, Methods, tables, figure legends, references, and supporting information, and have removed all visible reference-management placeholders such as “Error! Reference source not found.” The epidemiologic background, sleep-related literature, and Chinese Food Composition Table citation have all been corrected and linked to the appropriate numbered references. We also rechecked the reference list to ensure that the citations are numbered in the order of appearance and that the reference format follows the journal style. These corrections have been made throughout the revised manuscript.

***Point 2:*** *The analytic approach does not appear fully aligned with the matched design. The authors state that cases and controls were matched at a 1:2 ratio by age, sex, and residential proximity, yet the analysis is presented using standard multivariable logistic regression and conventional between-group significance testing in Table 1. For a matched case-control study, the authors should explain clearly why unconditional logistic regression was chosen instead of conditional logistic regression, and they should justify how the matching factors were handled analytically. At minimum, the statistical section should explicitly address whether the matching was preserved in the regression framework, whether any matched-set identifier was used, and whether sensitivity analyses with conditional logistic regression produce comparable estimates. Without that clarification, the current estimates are difficult to interpret with confidence.*

**Response 2:** We appreciate this important comment. In the revised manuscript, we have reanalyzed the data using multivariable conditional logistic regression to align the analysis with the 1:2 matched case-control design. All regression models were conditioned on matched sets, thereby preserving the matching structure of the study. We have revised the Statistical analyses section to explicitly state that conditional logistic regression was used and that all models were conditioned on matched sets. We also clarified in the Methods that cases and controls were individually matched by age and sex, and that controls were recruited from the same source population as cases to reduce selection bias. In addition, Table 1 is now presented only as a descriptive summary of participant characteristics, whereas the primary association analyses are based on conditional logistic regression.

***Point 3:*** *There are several concerns regarding confounding control and exposure assessment that require a more rigorous treatment. The final model adjusts for age, sex, education, income, family history, smoking, fish oil supplementation, diabetes, and BMI, yet important factors for lung cancer epidemiology appear insufficiently addressed, including total energy intake, alcohol use, passive smoking, occupational exposure, indoor air pollution, and other environmental carcinogen exposures. In addition, dietary intake and sleep behaviors were both assessed retrospectively for the prior year, with cases reporting the year before diagnosis and controls reporting the year before questionnaire completion. This design is inherently vulnerable to recall bias and reverse causation, especially because pre-diagnostic symptoms may affect appetite, food choices, sleep quality, snoring, daytime sleepiness, or body weight. These limitations should be discussed in a much more explicit and critical manner.*

**Response 3:** We thank the reviewer for this helpful suggestion. We have expanded the covariate adjustment in the fully adjusted model. In addition to age, sex, education level, household income, lung cancer family history, smoking status, fish oil supplementation, diabetes at baseline, and BMI, the revised Model 2 further adjusts for energy intake, alcohol intake frequency, passive smoking, occupational exposure, and hot cooking oil exposure.

We also revised the Methods to clarify the exposure assessment window: dietary intake and sleep behaviors were assessed with reference to the 12 months before lung cancer diagnosis for cases and the 12 months before questionnaire completion for controls. In addition, we expanded the Limitations section to discuss recall bias and reverse causation more explicitly. We now state that pre-diagnostic symptoms, including fatigue, appetite changes, and respiratory discomfort, may have affected participants’ dietary intake and sleep behaviors before formal diagnosis, raising the possibility of reverse causation.

***Point 4:*** *The numerical presentation and interpretation need tightening. The sample-size calculation indicated that at least 349 cases and 698 controls were needed, yet the final analytic sample consisted of 327 cases and 654 controls. The manuscript should state clearly that the final sample did not reach the planned target and discuss the implications for statistical power, particularly for stratified and interaction analyses. This is especially relevant because the paper places visible emphasis on subgroup findings across PUFA strata, while the figure legend itself states that no statistically significant multiplicative interaction was observed for sleep patterns and PUFA intake, with all P for interaction values greater than 0.05. In that setting, the Discussion and Abstract should be moderated substantially, because describing the association as “more evident” across certain strata may otherwise overstate what the data actually support.*

**Response 4:** We apologize for the unclear and inaccurate presentation of the sample-size calculation in the previous version of the manuscript, and we thank the reviewer for bringing this issue to our attention. We have carefully rechecked and clarified the sample-size calculation in the revised manuscript. Because our study was designed to examine the associations of sleep patterns and dietary PUFA intake with lung cancer in a matched case-control setting, we recalculated the required sample size by considering evidence from previous case-control studies on sleep and lung cancer, as well as epidemiological studies on PUFA intake and lung cancer. Based on these updated assumptions and a 1:2 case-to-control matching ratio, the minimum required sample size was 205 cases and 410 controls. After exclusions and matching, the final analytic sample included 322 cases and 644 matched controls, which exceeded the minimum requirement for the primary analyses.

We agree with the reviewer that the stratified and interaction analyses should be interpreted more cautiously. Therefore, we have moderated the Abstract and Discussion to avoid overstating the subgroup findings. In the revised manuscript, the stratified analyses are described as exploratory, and we explicitly state that formal tests for multiplicative interaction between sleep patterns and PUFA intake were not statistically significant. We have also revised the wording so that these findings are presented as possible stratified patterns rather than evidence of a definite interaction or effect modification.

***Point 5:*** *Several parts of the interpretation are too strong for an observational case-control analysis. The Conclusion currently states that “it is critical to improve” dietary PUFA intake and sleep quality and refers to “joint interventional measures aiming to prevent lung cancer.” That language is not justified by the present design, which can show association but not causation. This concern is amplified by the fact that cases, compared with controls, had lower diabetes prevalence and lower obesity prevalence, which is counterintuitive enough to warrant cautious interpretation and fuller discussion of possible selection effects or residual confounding. The Conclusions should therefore be rewritten in a more restrained epidemiologic manner, emphasizing association, uncertainty, and the need for prospective validation rather than implying preventive recommendations.*

**Response 5:** We agree with the reviewer. We have revised the Abstract Conclusion, Discussion, and final Conclusion to use more restrained epidemiologic language. We removed statements implying intervention, prevention, or causality, including phrases such as “it is critical to improve” and “joint interventional measures aiming to prevent lung cancer.” The revised conclusion now emphasizes that higher dietary omega-3 PUFA intake and favorable sleep patterns were associated with lower odds of lung cancer in this matched case-control study, and that these observational findings require confirmation in large-scale prospective studies. We also added a brief statement in the Limitations section noting that the lower prevalence of diabetes and obesity among cases than controls warrants cautious interpretation and may reflect selection effects or residual confounding.

***Point 6:*** *There is a formal reporting problem regarding data availability that should be corrected for journal compliance. In the submission materials, the authors selected “Yes - all data are fully available without restriction,” yet the written statement says that the datasets are available from the corresponding author on reasonable request. Those two statements are not equivalent, and the latter does not satisfy the former claim. This inconsistency should be resolved in a way that conforms exactly to the journal’s data-availability requirements. The same level of care is also needed for language editing more broadly, because the manuscript contains numerous awkward phrases and grammatical problems that reduce precision and readability.*

**Response 6:** We thank the reviewer for noting this inconsistency. We have revised the Data availability statement so that it is consistent with the submission selection. The revised statement indicates that the datasets used and/or analyzed during the current study are available within the manuscript and its supporting information files. We have also prepared the relevant supporting data file for journal submission to ensure compliance with the journal’s data-availability requirements.

In addition, we carefully edited the manuscript for grammar, precision, and readability. We revised awkward phrases, removed overly strong causal or interventional wording, improved consistency in terminology, and checked the manuscript for reference-management errors and formatting issues. These language revisions were made throughout the Abstract, Introduction, Methods, Results, Discussion, Conclusion, figure legend, and supplementary-related statements.

**Response to Reviewer 2 Comments**

***Point 1:* *Conceptual clarity and rationale***

*The manuscript attempts to link PUFAs, sleep, and lung cancer, but the underlying biological framework is weak. The discussion of inflammation and related mechanisms is superficial. A clearer rationale explaining how PUFA intake may interact with sleep related pathways in carcinogenesis is needed.*

**Response 1:** We thank the reviewer for this helpful comment. We agree that the biological rationale linking PUFA intake, sleep-related pathways, and lung cancer needed to be strengthened. In the revised Discussion, we clarified why omega-3 PUFAs may be particularly relevant to the observed stratified pattern, given their potential roles in inflammation, immune regulation, metabolism, and circadian biology. We also briefly acknowledged the biological complexity of omega-6 and total PUFAs. Throughout this section, we used cautious language to emphasize that these mechanisms remain hypothetical and were not directly tested in the present observational study.

***Point 2:*** ***Overinterpretation of interaction/stratified results***

*Although stratified analyses are presented, the interaction tests are not statistically significant. Despite this, the manuscript emphasizes differential associations across PUFA strata. This is misleading. These findings should be clearly presented as exploratory, and the conclusions should be toned down.*

**Response 2:** We agree with the reviewer. In the revised Results and Discussion, we explicitly state that formal tests for multiplicative interaction between sleep patterns and PUFA intake were not statistically significant. We therefore avoid interpreting the stratified findings as evidence of a definite interaction or effect modification.

We also revised both the Abstract Conclusion and the final Conclusion to use more cautious wording. Specifically, instead of emphasizing differential associations across PUFA strata, we now state that “the inverse sleep–lung cancer association was observed primarily/mainly among participants with higher omega-3 PUFA intake.” We further added cautious language indicating that these findings do not establish causality and warrant confirmation in prospective studies.

***Point 3:*** ***Framing of objectives***

*The study objectives are not clearly structured. The authors should distinguish between:*

*• primary analyses (independent associations), and*

*• secondary/exploratory analyses (stratification)*

**Response 3:** We thank the reviewer for this helpful suggestion. We have revised both the final paragraph of the Introduction and the Statistical analyses section to distinguish more clearly between the primary and secondary objectives. In the Introduction, we now state that “The primary objective of this study was to examine the associations of dietary PUFA intake and sleep patterns with lung cancer in a population from southwest China,” and that “As a secondary objective, we assessed whether the sleep–lung cancer association varied by dietary PUFA intake level.” Consistently, in the Statistical analyses section, we clarified that “The primary analyses examined the associations of dietary PUFA intake and sleep patterns separately with the odds of lung cancer,” whereas “Secondary exploratory analyses assessed whether the association between sleep patterns and lung cancer differed by level of dietary PUFA intake.”

***Point 4:* *Confounding (especially smoking)***

*Smoking is only adjusted as a categorical variable. Given its importance in lung cancer, more detailed adjustment (if available) or stratified analyses are recommended. This limitation should also be discussed more explicitly.*

**Response 4:** We appreciate this important comment. Smoking is a major risk factor for lung cancer, and we agree that careful control for smoking is essential. In the original analysis, smoking status had already been included as a covariate in Model 1 and was categorized as never smokers, current smokers, and former smokers. In the revised manuscript, this adjustment was retained, and we further expanded the fully adjusted model to include additional lung cancer-related covariates, including energy, passive smoking, occupational exposure, hot cooking oil exposure, and alcohol intake frequency, in order to reduce potential confounding as much as possible with the available data.

However, detailed quantitative smoking information, such as pack-years, was not available for all participants in the current dataset. Therefore, we were unable to adjust for pack-years or conduct more detailed smoking dose–response analyses. We have now acknowledged this limitation more explicitly in the Discussion: “Third, although we adjusted for a wide range of potential confounders, residual confounding may still remain from unmeasured or incompletely captured factors, such as pack-years of smoking, night-shift work, and specific dietary factors including red and processed meat intake”. We also revised the interpretation of the findings to avoid implying causality and to emphasize the need for future prospective studies.

***Point 5:*** ***Statistical issues***

*• Clarify whether conditional logistic regression was used given the matching design.*

*• Dichotomization of PUFA intake (median split) should be justified or supplemented*

*with more informative analyses (e.g., trends).*

**Response 5:** We thank the reviewer for raising these statistical issues. First, we have revised the Statistical analyses section to clarify that multivariable conditional logistic regression was used to account for the matched case-control design. All regression models were conditioned on matched sets, thereby preserving the matching structure.

Second, we have clarified the role of PUFA dichotomization. In the primary analyses, dietary omega-3, omega-6, and total PUFA intake were analyzed both as continuous variables and as tertiles, and tests for linear trend across tertiles were performed. The median split was used only for the secondary stratified analyses to define low- and high-intake groups, which allowed sufficient numbers of participants within each stratum. We have revised the Methods and Figure 2 legend to make this distinction clearer and have emphasized that the median-based stratified analyses were exploratory.

***Point 6:*** ***Strength of conclusions***

*The conclusions are too strong for a case–control study. Statements implying prevention should be revised to reflect observational associations only.*

**Response 6:** We agree with the reviewer. We have revised the Abstract Conclusion, Discussion, and final Conclusion to remove language that could imply prevention, intervention, or causality. Terms suggesting that dietary PUFA intake or sleep quality should be improved as preventive measures have been removed. The revised conclusions now state that higher dietary omega-3 PUFA intake and favorable sleep patterns were associated with lower odds of lung cancer in this matched case-control study. We also explicitly state that these observational findings do not establish causality and require confirmation in large-scale prospective studies.

***Minor comments***

*• Language and grammar need editing.*

*• Some repetition between sections.*

*• Methods (sleep score, exposure timing) need clearer description.*

**Response:** We thank the reviewer for these comments. We have edited the manuscript for language, grammar, readability, and precision, and reduced repetition between sections. We also revised the Methods section to clarify the healthy sleep score, including the five sleep characteristics, favorable-response definitions, and classification of sleep patterns. Finally, we clarified the exposure assessment window, specifying that dietary intake and sleep behaviors were assessed for the 12 months before lung cancer diagnosis for cases and for the 12 months before questionnaire completion for controls.
