# Supplementary material for: Associations of dietary polyunsaturated fatty acids and sleep patterns with lung cancer in southwest China: A case-control study
Source: PLoS One. 2026 Sep 30;21(9):e0358576. doi: 10.1371/journal.pone.0358576 (PMC13626354; doi:10.1371/journal.pone.0358576)
Supplement: Supplementary file 5 [file pone.0358576.s009.docx]

**Associations of dietary polyunsaturated fatty acids and sleep patterns with lung cancer in southwest China: a case-control study**

(Manuscript ID:PONE-D-26-10673)

Dear reviewers,

Thank you for your valuable comments. We have modified the manuscript, and revisions were highlighted in red to make sure changes could be easily visible to you. Please see our responses to the comments.

**Response to Reviewer 1 Comments**

***Point 1:*** *First, the manuscript still needs stricter causal restraint. Although the authors appropriately describe the work as a matched case-control study, several interpretive statements still imply prevention or risk reduction rather than association. Dietary intake and sleep patterns were assessed retrospectively, and lung cancer symptoms, prediagnostic illness, anxiety, cough, weight loss, appetite change, or smoking-related comorbidity may have influenced diet and sleep before recruitment. The authors should consistently use “associated with lower odds” rather than “reduced risk,” “protective effect,” or “preventive role,” unless clearly referring to hypotheses requiring confirmation in prospective cohorts.*

**Response 1:** Thank you for this important comment. We have revised the manuscript throughout to use more cautious wording, consistently describing our findings as “associated with lower odds of lung cancer” rather than implying risk reduction, prevention, or protective effects.

***Point 2:*** *Second, the case and control definitions require more transparent reporting. The manuscript should state clearly whether lung cancer cases were incident, newly diagnosed, pathologically confirmed, and recruited before treatment initiation. The source population for controls should also be described more rigorously, including whether controls came from the same catchment area, whether they were screened for cancer or major respiratory disease, and whether the same exclusion criteria were applied. A matched design is only convincing if case and control ascertainment are comparable. The current description remains too brief for judging selection bias.*

**Response 2:** Thank you for this important comment. We have revised the Study design and participants section to provide a clearer definition of both cases and controls. We now specify that cases were newly or recently diagnosed patients with a definite histopathological diagnosis of lung cancer (ICD-10 C33–C34). Because eligibility was based on a diagnosis within the previous 2 years, treatment status at recruitment was not consistently available and recruitment was not restricted to the pretreatment period. However, the questionnaire was designed to collect information on exposures and relevant conditions before lung cancer diagnosis. We also clarify that controls were recruited from the same geographic areas and randomly sampled communities as the cases, and that the same additional exclusion criteria were applied where applicable. Although information on prior individual screening for cancer or major respiratory disease was not available, eligible controls had no cancer record in the Chongqing Tumor Registration System and self-reported no current or previous history of cancer.

***Point 3:*** *Third, the dietary PUFA assessment needs stronger methodological detail. The authors used a validated semi-quantitative food frequency questionnaire, but the manuscript should provide more information on the validation population, recall period, food-composition database, energy-adjustment method, portion-size estimation, and whether PUFA intake was corrected for total energy using residual or density methods. Omega-3, omega-6, and total PUFA intake are likely correlated with total diet quality, fish intake, socioeconomic status, cooking practices, and health-conscious behaviors. Therefore, the interpretation should not isolate PUFA biology unless dietary-pattern confounding is better addressed.*

**Response 3:** Thank you for this helpful comment. We have expanded the Methods section to provide more detailed information on dietary PUFA assessment. Specifically, we clarified that dietary intake was assessed using an SQFFQ developed from 3-day weighed food records in Chongqing adults, with relative validity evaluated in 92 healthy adults aged 35–55 years from urban and rural areas of Chongqing and reproducibility assessed in 48 adults of the same age range using repeated administrations at a 4-week interval. We also added details on the food-composition database, specifying that nutrient and energy intakes were estimated using the Chinese Food Composition Tables (Standard Edition, 6th Edition), and that daily intakes of omega-3, omega-6, and total PUFAs were calculated by summing their contributions from all reported food items and expressed as grams per day.

Omega-3, omega-6, and total PUFA intakes were not energy-adjusted using residual or nutrient-density methods; instead, total energy intake was included as a covariate in the multivariable models. In the Discussion, we revised the interpretation to avoid isolating PUFA-specific biological effects and emphasized that potential biological explanations should be considered in the context of broader dietary and lifestyle patterns. We have made every effort to consider all relevant factors, but it is possible that some factors have not been fully accounted for; these are described in the Limitations section.

***Point 4:*** *Fourth, the sleep-pattern analysis is clinically interesting but still insufficiently decomposed. The composite healthy sleep score based on five sleep characteristics is useful, but the manuscript should report the association of each individual sleep component with lung cancer odds, including sleep duration, chronotype, insomnia symptoms, snoring, and daytime sleepiness or equivalent components. A composite score may hide opposing effects across components. The authors should also clarify whether sleep was assessed for the period before diagnosis and whether cancer-related symptoms could have affected reported sleep among cases.*

**Response 4:** Thank you for this important comment. We have added component-specific analyses for each of the five sleep characteristics, including sleep duration, chronotype, insomnia, snoring, and daytime sleepiness, with the results presented in S1 Table. Compared with definite evening types, definite morning types, more morning than evening types, and more evening than morning types were associated with lower odds of lung cancer across all models, whereas no overall associations were observed for the other sleep characteristics.

For cases, all sleep characteristics referred to the period before lung cancer diagnosis. Although the cases had already been diagnosed at the time of data collection, prediagnostic sleep characteristics were obtained retrospectively through participant recall. The potential for recall bias has been acknowledged in the Limitations section.

***Point 5:*** *Fifth, the statistical interpretation of stratified and interaction analyses should be softened. The authors report that the inverse sleep–lung cancer association was observed primarily among participants with higher omega-3 PUFA intake, but median-based dichotomization of PUFA intake can reduce information and increase instability. The manuscript should report the exact P values for multiplicative interaction between sleep pattern and each PUFA class, as well as stratum-specific sample sizes and confidence intervals. Without a statistically significant interaction, the authors should not imply effect modification. Multiple comparisons across continuous models, tertiles, sleep categories, PUFA strata, and interactions also require more cautious wording.*

**Response 5:** We thank the reviewer for this helpful comment. We have revised the Results section, Fig 2, and its legend accordingly. Fig 2 now presents the stratum-specific numbers of cases and total participants, together with the corresponding ORs and 95% CIs. In the Results section, we report the exact *P* values for the multiplicative interaction terms between sleep pattern and each PUFA class. For each PUFA class, two cross-product terms were tested, corresponding to medium sleep pattern × higher PUFA intake and favorable sleep pattern × higher PUFA intake, with unfavorable sleep pattern and lower PUFA intake as the reference categories. None of the interaction terms was statistically significant.

Accordingly, we have revised the interpretation of the stratified analyses and now describe them as exploratory. We no longer imply effect modification based solely on differences in stratum-specific estimates in the absence of statistically significant interaction. We have also expanded the Limitations section to acknowledge that dichotomizing PUFA intake at the median may lead to information loss and less stable stratum-specific estimates. These revisions emphasize that the stratified findings should be interpreted cautiously and should not be considered definitive evidence of interaction.

***Point 6:*** *Sixth, the manuscript must cite additional recent literature to improve contextualization within nutritional epidemiology, omega-3-related disease prevention, metabolic inflammation, oxidative stress, and observational evidence interpretation. The following four studies should be cited: 1. PMID: 41482231; doi: 10.1016/j.tjnut.2025.101289, because it provides a nutrition-focused systematic review and meta-analysis on omega-3 fatty acids as a preventive strategy and is directly relevant to framing omega-3 exposure, prevention claims, and evidence limitations; 2. PMID: 40987202; doi: 10.1016/j.jnha.2025.100632, because it evaluates omega-3 intake in relation to diabetic microvascular outcomes and helps contextualize dietary PUFA intake as a systemic metabolic and inflammatory exposure; 3. PMID: 41935442; doi: 10.1016/j.redox.2025.103976, because mitochondrial dysfunction and oxidative stress are relevant to mechanistic discussion of diet, inflammation, sleep disruption, and carcinogenesis; 4. PMID: 41038126; doi: 10.1016/j.jdiacomp.2025.109178, because lipid-modifying and metabolic intervention evidence provides useful translational context for interpreting lipid-related exposures and disease outcomes without overstating causality.*

**Response 6:** Thank you for this valuable suggestion. We have added all four recommended studies in the Discussion section. These references were incorporated to strengthen the contextualization of omega-3 fatty acids in disease prevention, systemic metabolic and inflammatory processes, mitochondrial dysfunction and oxidative stress, and the cautious interpretation of lipid-related observational findings without overstating causality.

***Point 7:*** *The manuscript also requires final PLOS-specific compliance and language correction. The Data Availability statement says that all data are fully available and later states that datasets are available within the manuscript and supporting information files; the authors should ensure that the minimal anonymized dataset and analytic code are actually supplied in Supporting Information or a repository. The funding statement should include author initials, grant numbers, full funder names, funder URLs, and a clear statement on whether the funders had any role in study design, data collection, analysis, decision to publish, or manuscript preparation. The manuscript should also standardize PUFA, SQFFQ, OR, CI, BMI, Model 1, Model 2, passive smoking, occupational exposure, hot cooking oil exposure, healthy sleep score, and conditional logistic regression terminology. Minor grammar and formatting issues remain, including spacing before references and inconsistent use of “southwest China” versus “southwest, China.”*

**Response 7:** Thank you for this careful review of the manuscript’s PLOS-specific compliance and language consistency. We have now provided the minimal anonymized dataset and the corresponding analytic code in the Supporting Information. In accordance with the journal’s instruction that funding information should not appear in the manuscript itself, the funding details were entered in the online submission form rather than included in the manuscript. The submission form includes the relevant author initials, grant numbers, full funder names, and funder information. We also stated that the funders had no role in the study design, data collection and analysis, decision to publish, or preparation of the manuscript in the cover letter. Finally, we standardized the use of PUFA, SQFFQ, OR, CI, BMI, Model 1, Model 2, passive smoking, occupational exposure, hot cooking oil exposure, healthy sleep score, and conditional logistic regression throughout the manuscript. We also performed a final language and formatting check and corrected the remaining grammatical, spacing, and consistency issues, including the use of “southwest China.”

**Response to Reviewer 2 Comments**

***Point 1:*** *The authors have acknowledged the limitation regarding smoking adjustment in the Discussion. However, considering the major role of smoking in lung cancer, this limitation should be emphasized slightly more. If detailed smoking information (e.g., pack-years) is available, its inclusion would strengthen the analysis. If not, this should be clearly stated as a limitation.*

**Response 1:** Thank you for this important comment. We fully agree that smoking is the dominant established risk factor for lung cancer and that insufficient adjustment for cumulative smoking exposure may lead to residual confounding. In the present study, we adjusted for active smoking status, categorized as never, current, and former smokers, and additionally adjusted for passive smoking exposure in the fully adjusted model. However, detailed cumulative smoking exposure data, including pack-years, smoking duration, cigarettes smoked per day, and smoking cessation history, were not collected. Therefore, we were unable to further adjust for smoking intensity or duration. In response to this comment, we have revised the limitation section of the Discussion to more clearly acknowledge this issue.

***Point 2:*** *For omega-6 and total PUFAs, the continuous analyses were not statistically significant after full adjustment, whereas the tertile analyses remained significant. A brief explanation for this difference (such as possible non-linear associations or threshold effects) would improve the discussion.*

**Response 2:** Thank you for this helpful suggestion. We agree that the discrepancy between the continuous and tertile-based analyses for omega-6 and total PUFAs requires further clarification. This difference may partly reflect a non-linear or threshold-type association, as the continuous model assumes a constant linear association per 1-g/day increment, whereas tertile-based analyses compare broader intake categories and may better capture contrasts between relatively low and high intake groups; residual confounding and differences in model specification may also contribute to this discrepancy. In response to this comment, we have revised the Discussion to briefly explain this possibility and to emphasize that the tertile-based findings for omega-6 and total PUFAs should be interpreted with caution.

***Point 3:*** *The reported registration date appears to be later than the study period. The authors should clarify whether the study was registered retrospectively.*

**Response 3:** Thank you for pointing this out. We confirm that the study was retrospectively registered. This was an observational matched case-control study rather than an interventional clinical trial. Participant recruitment and data collection were conducted from August 22, 2023, to September 30, 2024, whereas the study was registered in the Chinese Clinical Trial Registry on July 31, 2025. Therefore, the registration date was later than the study period.

We would like to clarify that the study had obtained ethical approval before and during the conduct of the research. In addition to the original ethics approval, an ethics approval extension review was completed by the Ethics Committee of Chongqing Center for Disease Control and Prevention, and approval to continue the study was granted. The retrospective registration did not affect the study design, participant recruitment, data collection, or statistical analysis.

In response to this comment, we have revised the manuscript to explicitly state that the study was retrospectively registered and to clarify the timing of registration relative to the study period.

***Point 4:*** *The overall language has improved. However, a final proofreading would help to correct a few minor grammatical errors and improve readability.*

**Response 4:** Thank you for your positive comment. We have conducted a final proofreading of the manuscript and corrected minor grammatical errors, formatting inconsistencies, and readability issues throughout the text, tables, and figure legend.
