# Supplementary material for: Associations of dietary polyunsaturated fatty acids and sleep patterns with lung cancer in southwest China: A case-control study
Source: PLoS One. 2026 Sep 30;21(9):e0358576. doi: 10.1371/journal.pone.0358576 (PMC13626354; doi:10.1371/journal.pone.0358576)
Supplement: Supplementary file 6 [file pone.0358576.s010.docx]

**Associations of dietary polyunsaturated fatty acids and sleep patterns with lung cancer in southwest China: a case-control study**

(Manuscript ID:PONE-D-26-10673)

Dear Editors and Reviewers,

Thank you for your valuable comments and suggestions. We have carefully revised the manuscript, and all changes are highlighted in red for ease of review.

**Response to Editors’ Comments**

***Point 1:*** *Explicitly acknowledging the lack of cumulative smoking exposure data (pack-years) as a limitation.*

**Response 1:** Thank you for this important comment. We have explicitly acknowledged this issue in the Limitations section and clarified that residual confounding related to the lack of detailed cumulative smoking exposure data cannot be excluded.

***Point 2:*** *Expanding the discussion slightly regarding the non-linear/threshold dynamics observed in omega-6 and total PUFA tertile models vs. continuous models.* **Response 2:** Thank you for this helpful suggestion. We have expanded the Discussion to clarify the possible reasons for the discrepancy between continuous and tertile-based analyses of omega-6 and total PUFA intake. We now discuss that continuous models assume an approximately constant change in the log odds of lung cancer per unit increase across the intake range, whereas tertile-based analyses may reveal potential non-linear or threshold/plateau patterns.

***Point 3:*** *Clarifying study registry status and retrospective timing, if applicable.* **Response 3:** Thank you for this comment. We have clarified the registration status and timing in the Methods section, including the registry ID, registration date, and retrospective nature of the registration.

***Point 4:*** *Correcting a minor typesetting error in the abstract.*

**Response 4:** Thank you for noting this issue. We carefully rechecked the Abstract and corrected the identified inconsistency. We also rechecked the numerical results reported in the Abstract and the main text to ensure consistency with the corresponding tables and final statistical analyses.

**Response to Reviewers’ Comments**

***Point 1:*** *Smoking Confounding & Pack-Years (Limitation): While adjusting for categorical smoking status (never/current/former) and passive smoking is appreciated, smoking remains the predominant risk factor for lung cancer. If cumulative smoking exposure (e.g., pack-years or daily intensity/duration) was not collected, please explicitly state this in the Limitations section as a potential source of residual confounding.*

**Response 1:** Thank you for this important observation. We agree that adjustment for categorical smoking status cannot fully account for differences in cumulative tobacco exposure. We have therefore explicitly stated in the Limitations section that detailed cumulative smoking exposure data, including pack-years, smoking duration, cigarettes smoked per day, and smoking cessation history, were unavailable. Although smoking status and passive smoking were included in the multivariable models, residual confounding by smoking intensity and cumulative exposure cannot be excluded.

***Point 2:*** *Discussion of Continuous vs. Tertile PUFA Findings: In the results, continuous analyses for omega-6 and total PUFA intake were non-significant after full adjustment, whereas tertile-based analyses showed significant inverse associations. Please add a brief paragraph in the Discussion addressing this discrepancy (e.g., discussing potential non-linear relationships, threshold/plateau effects, or narrow intake ranges).* **Response 2:** Thank you for this helpful comment. We have expanded the Discussion to clarify the possible reasons for the discrepancy between continuous and tertile-based analyses of omega-6 and total PUFA intake. We now discuss that continuous models assume an approximately constant change in the log odds of lung cancer per unit increase across the intake range, whereas tertile-based analyses may reveal potential non-linear or threshold/plateau patterns. We have also emphasized that these findings should be interpreted cautiously and do not establish a definitive threshold effect.

***Point 3:*** *Study Registration: Please clarify in the Methods section whether the study was registered in a clinical/observational study registry, and explicitly state if registration was retrospective relative to the study recruitment period (August 2023 – September 2024), including the registration ID and date.*

**Response 3:** Thank you for this comment. We have now explicitly provided this information in the Methods section. This observational matched case-control study was retrospectively registered in the Chinese Clinical Trial Registry (registration ID: ChiCTR2500106928) on July 31, 2025. Because participant recruitment and data collection occurred from August 22, 2023, to September 30, 2024, the registration took place after completion of recruitment and was therefore retrospective.
