# Supplementary material for: Associations of dietary polyunsaturated fatty acids and sleep patterns with lung cancer in southwest China: A case-control study
Source: PLoS One. 2026 Sep 30;21(9):e0358576. doi: 10.1371/journal.pone.0358576 (PMC13626354; doi:10.1371/journal.pone.0358576)
Supplement: Peer review history

# Peer review: Decision Letter 0

**DOI:** 10.1371/journal.pone.0358576.r001

**Authors:** Assoc. Prof. Phakkharawat Sittiprapaporn

19 Apr 2026

Dear Dr. Chen,

Thank you for submitting your manuscript to PLOS ONE. After careful consideration, we feel that it has merit but does not fully meet PLOS ONE’s publication criteria as it currently stands. Therefore, we invite you to submit a revised version of the manuscript that addresses the points raised during the review process.

Please submit your revised manuscript by Jun 03 2026 11:59PM. If you will need more time than this to complete your revisions, please reply to this message or contact the journal office at plosone@plos.org. When you're ready to submit your revision, log on to https://www.editorialmanager.com/pone/ and select the 'Submissions Needing Revision' folder to locate your manuscript file.

- A letter that responds to each point raised by the academic editor and reviewer(s). You should upload this letter as a separate file labeled 'Response to Reviewers'.
- A marked-up copy of your manuscript that highlights changes made to the original version. You should upload this as a separate file labeled 'Revised Manuscript with Track Changes'.
- An unmarked version of your revised paper without tracked changes. You should upload this as a separate file labeled 'Manuscript'.

If you would like to make changes to your financial disclosure, please include your updated statement in your cover letter. Guidelines for resubmitting your figure files are available below the reviewer comments at the end of this letter.

If applicable, we recommend that you deposit your laboratory protocols in protocols.io to enhance the reproducibility of your results. Protocols.io assigns your protocol its own identifier (DOI) so that it can be cited independently in the future. For instructions see: https://journals.plos.org/plosone/s/submission-guidelines#loc-laboratory-protocols. Additionally, PLOS ONE offers an option for publishing peer-reviewed Lab Protocol articles, which describe protocols hosted on protocols.io. Read more information on sharing protocols at https://plos.org/protocols?utm_medium=editorial-email&utm_source=authorletters&utm_campaign=protocols.

As the corresponding author, your ORCID iD is verified in the submission system and will appear in the published article. PLOS supports the use of ORCID, and we encourage all coauthors to register for an ORCID iD and use it as well. Please encourage your coauthors to verify their ORCID iD within the submission system before final acceptance, as unverified ORCID iDs will not appear in the published article. *Only*  the individual author can complete the verification step; PLOS staff *cannot*  verify ORCID iDs on behalf of authors.

We look forward to receiving your revised manuscript.

Kind regards,

Assoc. Prof. Phakkharawat Sittiprapaporn, Ph.D.

Academic Editor

PLOS One

**Journal Requirements:**

When submitting your revision, we need you to address these additional requirements.

1. Please ensure that your manuscript meets PLOS ONE's style requirements, including those for file naming. The PLOS ONE style templates can be found at

https://journals.plos.org/plosone/s/file?id=wjVg/PLOSOne_formatting_sample_main_body.pdf and

https://journals.plos.org/plosone/s/file?id=ba62/PLOSOne_formatting_sample_title_authors_affiliations.pdf

2. Thank you for stating the following financial disclosure:

“This work was funded by the Natural Science Foundation Project of Chongqing, Chongqing Science and Technology Commission (No.CSTB2024NSCQ-KJFZMSX0027), and the Science-Health Joint Medical Scientific Research Project of Chongqing (NO.2023MSXM064).”

Please state what role the funders took in the study.  If the funders had no role, please state: "The funders had no role in study design, data collection and analysis, decision to publish, or preparation of the manuscript."

If this statement is not correct you must amend it as needed.

Please include this amended Role of Funder statement in your cover letter; we will change the online submission form on your behalf.

3. Please note that funding information should not appear in any section or other areas of your manuscript. We will only publish funding information present in the Funding Statement section of the online submission form. Please remove any funding-related text from the manuscript.

4. In the online submission form you indicate that your data is not available for proprietary reasons and have provided a contact point for accessing this data. Please note that your current contact point is a co-author on this manuscript. According to our Data Policy, the contact point must not be an author on the manuscript and must be an institutional contact, ideally not an individual. Please revise your data statement to a non-author institutional point of contact, such as a data access or ethics committee, and send this to us via return email. Please also include contact information for the third party organization, and please include the full citation of where the data can be found.

5. Your ethics statement should only appear in the Methods section of your manuscript. If your ethics statement is written in any section besides the Methods, please move it to the Methods section and delete it from any other section. Please ensure that your ethics statement is included in your manuscript, as the ethics statement entered into the online submission form will not be published alongside your manuscript.

6. Please include captions for your Supporting Information files at the end of your manuscript, and update any in-text citations to match accordingly. Please see our Supporting Information guidelines for more information: http://journals.plos.org/plosone/s/supporting-information.

7. If the reviewer comments include a recommendation to cite specific previously published works, please review and evaluate these publications to determine whether they are relevant and should be cited. There is no requirement to cite these works unless the editor has indicated otherwise.

Reviewers' comments:

Reviewer's Responses to Questions

**Comments to the Author**

1. Is the manuscript technically sound, and do the data support the conclusions?

Reviewer #1: Yes

Reviewer #2: Partly

**********

2. Has the statistical analysis been performed appropriately and rigorously?

Reviewer #1: Yes

Reviewer #2: Yes

**********

3. Have the authors made all data underlying the findings in their manuscript fully available?

The PLOS Data policy

Reviewer #1: Yes

Reviewer #2: Yes

**********

4. Is the manuscript presented in an intelligible fashion and written in standard English?

Reviewer #1: Yes

Reviewer #2: Yes

**********

Reviewer #1: After carefully reading the manuscript, I find that the topic is potentially of interest and the case-control dataset is reasonably sized, with 327 cases and 654 matched controls included in the final analysis. However, the present version still contains several important methodological, reporting, and interpretative deficiencies that should be corrected before the work can be considered further. My recommendation is minor revision, but the revision should be careful and substantive rather than merely cosmetic.

First, the manuscript contains obvious reference-management and manuscript-preparation errors that are not acceptable in its current form. The Introduction includes visible placeholders such as “Error! Reference source not found.” in the epidemiologic background and again in the sleep-related literature section, and a similar citation error appears for the Chinese Food Composition Table in the Methods. These errors are not trivial because they interrupt the scientific argument, obscure the evidentiary basis for key statements, and create concern regarding the overall care taken in manuscript preparation. These issues should be corrected throughout the full text, tables, figures, and supplementary materials before any further consideration.

Second, the analytic approach does not appear fully aligned with the matched design. The authors state that cases and controls were matched at a 1:2 ratio by age, sex, and residential proximity, yet the analysis is presented using standard multivariable logistic regression and conventional between-group significance testing in Table 1. For a matched case-control study, the authors should explain clearly why unconditional logistic regression was chosen instead of conditional logistic regression, and they should justify how the matching factors were handled analytically. At minimum, the statistical section should explicitly address whether the matching was preserved in the regression framework, whether any matched-set identifier was used, and whether sensitivity analyses with conditional logistic regression produce comparable estimates. Without that clarification, the current estimates are difficult to interpret with confidence.

Third, there are several concerns regarding confounding control and exposure assessment that require a more rigorous treatment. The final model adjusts for age, sex, education, income, family history, smoking, fish oil supplementation, diabetes, and BMI, yet important factors for lung cancer epidemiology appear insufficiently addressed, including total energy intake, alcohol use, passive smoking, occupational exposure, indoor air pollution, and other environmental carcinogen exposures. In addition, dietary intake and sleep behaviors were both assessed retrospectively for the prior year, with cases reporting the year before diagnosis and controls reporting the year before questionnaire completion. This design is inherently vulnerable to recall bias and reverse causation, especially because pre-diagnostic symptoms may affect appetite, food choices, sleep quality, snoring, daytime sleepiness, or body weight. These limitations should be discussed in a much more explicit and critical manner.

Fourth, the numerical presentation and interpretation need tightening. The sample-size calculation indicated that at least 349 cases and 698 controls were needed, yet the final analytic sample consisted of 327 cases and 654 controls. The manuscript should state clearly that the final sample did not reach the planned target and discuss the implications for statistical power, particularly for stratified and interaction analyses. This is especially relevant because the paper places visible emphasis on subgroup findings across PUFA strata, while the figure legend itself states that no statistically significant multiplicative interaction was observed for sleep patterns and PUFA intake, with all P for interaction values greater than 0.05. In that setting, the Discussion and Abstract should be moderated substantially, because describing the association as “more evident” across certain strata may otherwise overstate what the data actually support.

Fifth, several parts of the interpretation are too strong for an observational case-control analysis. The Conclusion currently states that “it is critical to improve” dietary PUFA intake and sleep quality and refers to “joint interventional measures aiming to prevent lung cancer.” That language is not justified by the present design, which can show association but not causation. This concern is amplified by the fact that cases, compared with controls, had lower diabetes prevalence and lower obesity prevalence, which is counterintuitive enough to warrant cautious interpretation and fuller discussion of possible selection effects or residual confounding. The Conclusions should therefore be rewritten in a more restrained epidemiologic manner, emphasizing association, uncertainty, and the need for prospective validation rather than implying preventive recommendations.

Sixth, there is a formal reporting problem regarding data availability that should be corrected for journal compliance. In the submission materials, the authors selected “Yes - all data are fully available without restriction,” yet the written statement says that the datasets are available from the corresponding author on reasonable request. Those two statements are not equivalent, and the latter does not satisfy the former claim. This inconsistency should be resolved in a way that conforms exactly to the journal’s data-availability requirements. The same level of care is also needed for language editing more broadly, because the manuscript contains numerous awkward phrases and grammatical problems that reduce precision and readability.

With regard to additional literature, a few literature may be considered if the authors wish to broaden the contextual discussion around nutritional fatty acids and chronic-disease associations. In particular, the following appear to be the most appropriate: 1. PMID: 41482231. DOI: 10.1016/j.tjnut.2025.101289. This may be relevant as an example of systematic synthesis on omega-3 exposure and disease risk. 2. PMID: 40987202. DOI: 10.1016/j.jnha.2025.100632. This is potentially useful because it is directly focused on dietary omega-3 intake as an exposure. 3. PMID: 40005319. DOI: 10.3390/medicina61020202. This may be cited only as a broader example of quantitative synthesis in chronic-disease epidemiology. 4. PMID: 39595078. DOI: 10.3390/biomedicines12112512. This may be considered if the authors wish to expand discussion of metabolic and oxidative-stress mechanisms.

Overall, the manuscript has merit and the core finding may be of interest to readers, but the present version still contains notable deficiencies in numerical consistency, methodological description, statistical presentation, interpretation of interaction results, data-availability compliance, and language quality. I therefore recommend minor revision. The work could become suitable for publication after careful correction of the points above.

Reviewer #2: This is a relevant study examining dietary PUFAs, sleep patterns, and lung cancer risk in a case–control design. The sample size and overall approach are acceptable. However, the manuscript in its current form lacks conceptual clarity and tends to overinterpret the findings, particularly regarding interaction effects.

The combination of diet, sleep, and cancer is potentially interesting, but the biological rationale—especially from an inflammation perspective—is not sufficiently developed, making the study feel somewhat unfocused.

Major Comment

1. Conceptual clarity and rationale

The manuscript attempts to link PUFAs, sleep, and lung cancer, but the underlying biological framework is weak. The discussion of inflammation and related mechanisms is superficial. A clearer rationale explaining how PUFA intake may interact with sleep-related pathways in carcinogenesis is needed.

2. Overinterpretation of interaction/stratified results

Although stratified analyses are presented, the interaction tests are not statistically significant. Despite this, the manuscript emphasizes differential associations across PUFA strata. This is misleading. These findings should be clearly presented as exploratory, and the conclusions should be toned down.

3. Framing of objectives

The study objectives are not clearly structured. The authors should distinguish between:

• primary analyses (independent associations), and

• secondary/exploratory analyses (stratification)

4. Confounding (especially smoking)

Smoking is only adjusted as a categorical variable. Given its importance in lung cancer, more detailed adjustment (if available) or stratified analyses are recommended. This limitation should also be discussed more explicitly.

5. Statistical issues

• Clarify whether conditional logistic regression was used given the matching design.

• Dichotomization of PUFA intake (median split) should be justified or supplemented with more informative analyses (e.g., trends).

6. Strength of conclusions

The conclusions are too strong for a case–control study. Statements implying prevention should be revised to reflect observational associations only.

Minor comments

• Language and grammar need editing.

• Some repetition between sections.

• Methods (sleep score, exposure timing) need clearer description.

Final Recommendation-Major Revision

The study is potentially publishable, but substantial revision is required to improve conceptual coherence and align interpretation with the data.

**********

what does this mean?). If published, this will include your full peer review and any attached files.

If you choose “no”, your identity will remain anonymous but your review may still be made public.

**Do you want your identity to be public for this peer review?**  For information about this choice, including consent withdrawal, please see our Privacy Policy

Reviewer #1: No

Reviewer #2: No

**********

[NOTE: If reviewer comments were submitted as an attachment file, they will be attached to this email and accessible via the submission site. Please log into your account, locate the manuscript record, and check for the action link "View Attachments". If this link does not appear, there are no attachment files.]

To ensure your figures meet our technical requirements, please review our figure guidelines: https://journals.plos.org/plosone/s/figures

You may also use PLOS’s free figure tool, NAAS, to help you prepare publication quality figures: https://journals.plos.org/plosone/s/figures#loc-tools-for-figure-preparation.

NAAS will assess whether your figures meet our technical requirements by comparing each figure against our figure specifications.

Attachment Submitted filename: Reviewer Report.pdf

# Author comment: Author response to Decision Letter 1

**DOI:** 10.1371/journal.pone.0358576.r002

15 Jun 2026

Dear Reviewers and Editor,

We have revised our paper according to each of the comments, and revisions were highlighted in red to make sure changes could be easily visible to you and reviewers. We have uploaded 'Response to Reviewers' file to explain point-by-point the details of the revisions in the manuscript and our responses to the reviewers' comments.We have also addressed the journal’s additional requirements in the revised manuscript and cover letter as appropriate.

Attachment Submitted filename: Response to reviewers.docx

# Peer review: Decision Letter 1

**DOI:** 10.1371/journal.pone.0358576.r003

**Authors:** Assoc. Prof. Phakkharawat Sittiprapaporn

26 Jun 2026

Dear Dr. Chen,

Thank you for submitting your manuscript to PLOS ONE. After careful consideration, we feel that it has merit but does not fully meet PLOS ONE’s publication criteria as it currently stands. Therefore, we invite you to submit a revised version of the manuscript that addresses the points raised during the review process.

Please submit your revised manuscript by Aug 10 2026 11:59PM. If you will need more time than this to complete your revisions, please reply to this message or contact the journal office at plosone@plos.org. When you're ready to submit your revision, log on to https://www.editorialmanager.com/pone/ and select the 'Submissions Needing Revision' folder to locate your manuscript file.

- A letter that responds to each point raised by the academic editor and reviewer(s). You should upload this letter as a separate file labeled 'Response to Reviewers'.
- A marked-up copy of your manuscript that highlights changes made to the original version. You should upload this as a separate file labeled 'Revised Manuscript with Track Changes'.
- An unmarked version of your revised paper without tracked changes. You should upload this as a separate file labeled 'Manuscript'.

If you would like to make changes to your financial disclosure, please include your updated statement in your cover letter. Guidelines for resubmitting your figure files are available below the reviewer comments at the end of this letter.

If applicable, we recommend that you deposit your laboratory protocols in protocols.io to enhance the reproducibility of your results. Protocols.io assigns your protocol its own identifier (DOI) so that it can be cited independently in the future. For instructions see: https://journals.plos.org/plosone/s/submission-guidelines#loc-laboratory-protocols. Additionally, PLOS ONE offers an option for publishing peer-reviewed Lab Protocol articles, which describe protocols hosted on protocols.io. Read more information on sharing protocols at https://plos.org/protocols?utm_medium=editorial-email&utm_source=authorletters&utm_campaign=protocols.

As the corresponding author, your ORCID iD is verified in the submission system and will appear in the published article. PLOS supports the use of ORCID, and we encourage all coauthors to register for an ORCID iD and use it as well. Please encourage your coauthors to verify their ORCID iD within the submission system before final acceptance, as unverified ORCID iDs will not appear in the published article. *Only*  the individual author can complete the verification step; PLOS staff *cannot*  verify ORCID iDs on behalf of authors.

We look forward to receiving your revised manuscript.

Kind regards,

Assoc. Prof. Phakkharawat Sittiprapaporn, Ph.D.

Academic Editor

PLOS One

Journal Requirements:

If the reviewer comments include a recommendation to cite specific previously published works, please review and evaluate these publications to determine whether they are relevant and should be cited. There is no requirement to cite these works unless the editor has indicated otherwise.

Reviewers' comments:

Reviewer's Responses to Questions

**Comments to the Author**

Reviewer #1: (No Response)

Reviewer #2: All comments have been addressed

**********

2. Is the manuscript technically sound, and do the data support the conclusions?

Reviewer #1: (No Response)

Reviewer #2: Yes

**********

3. Has the statistical analysis been performed appropriately and rigorously?

Reviewer #1: (No Response)

Reviewer #2: Yes

**********

4. Have the authors made all data underlying the findings in their manuscript fully available?

The PLOS Data policy

Reviewer #1: (No Response)

Reviewer #2: Yes

**********

5. Is the manuscript presented in an intelligible fashion and written in standard English?

Reviewer #1: (No Response)

Reviewer #2: Yes

**********

Reviewer #1: The manuscript entitled “Associations of dietary polyunsaturated fatty acids and sleep patterns with lung cancer in southwest China: a case-control study” examines dietary omega-3, omega-6, and total polyunsaturated fatty acid intake, composite sleep patterns, and lung cancer odds in a matched case-control study including 322 lung cancer cases and 644 matched controls from Chongqing, southwest China. The authors report that higher omega-3 PUFA intake was inversely associated with lung cancer in continuous analysis (OR = 0.82; 95% CI: 0.70–0.96), that the highest tertiles of omega-3, omega-6, and total PUFA intake were associated with lower lung cancer odds, and that favorable sleep patterns were associated with lower lung cancer odds compared with unfavorable sleep patterns. The revised manuscript is improved and the topic is suitable for PLOS ONE, but the present version still requires minor revision because several important issues in causal wording, exposure assessment, sleep-pattern interpretation, confounding control, interaction analysis, and PLOS reporting remain insufficiently controlled.

First, the manuscript still needs stricter causal restraint. Although the authors appropriately describe the work as a matched case-control study, several interpretive statements still imply prevention or risk reduction rather than association. Dietary intake and sleep patterns were assessed retrospectively, and lung cancer symptoms, prediagnostic illness, anxiety, cough, weight loss, appetite change, or smoking-related comorbidity may have influenced diet and sleep before recruitment. The authors should consistently use “associated with lower odds” rather than “reduced risk,” “protective effect,” or “preventive role,” unless clearly referring to hypotheses requiring confirmation in prospective cohorts.

Second, the case and control definitions require more transparent reporting. The manuscript should state clearly whether lung cancer cases were incident, newly diagnosed, pathologically confirmed, and recruited before treatment initiation. The source population for controls should also be described more rigorously, including whether controls came from the same catchment area, whether they were screened for cancer or major respiratory disease, and whether the same exclusion criteria were applied. A matched design is only convincing if case and control ascertainment are comparable. The current description remains too brief for judging selection bias.

Third, the dietary PUFA assessment needs stronger methodological detail. The authors used a validated semi-quantitative food frequency questionnaire, but the manuscript should provide more information on the validation population, recall period, food-composition database, energy-adjustment method, portion-size estimation, and whether PUFA intake was corrected for total energy using residual or density methods. Omega-3, omega-6, and total PUFA intake are likely correlated with total diet quality, fish intake, socioeconomic status, cooking practices, and health-conscious behaviors. Therefore, the interpretation should not isolate PUFA biology unless dietary-pattern confounding is better addressed.

Fourth, the sleep-pattern analysis is clinically interesting but still insufficiently decomposed. The composite healthy sleep score based on five sleep characteristics is useful, but the manuscript should report the association of each individual sleep component with lung cancer odds, including sleep duration, chronotype, insomnia symptoms, snoring, and daytime sleepiness or equivalent components. A composite score may hide opposing effects across components. The authors should also clarify whether sleep was assessed for the period before diagnosis and whether cancer-related symptoms could have affected reported sleep among cases.

Fifth, the statistical interpretation of stratified and interaction analyses should be softened. The authors report that the inverse sleep–lung cancer association was observed primarily among participants with higher omega-3 PUFA intake, but median-based dichotomization of PUFA intake can reduce information and increase instability. The manuscript should report the exact P values for multiplicative interaction between sleep pattern and each PUFA class, as well as stratum-specific sample sizes and confidence intervals. Without a statistically significant interaction, the authors should not imply effect modification. Multiple comparisons across continuous models, tertiles, sleep categories, PUFA strata, and interactions also require more cautious wording.

Sixth, the manuscript must cite additional recent literature to improve contextualization within nutritional epidemiology, omega-3-related disease prevention, metabolic inflammation, oxidative stress, and observational evidence interpretation. The following four studies should be cited: 1. PMID: 41482231; doi: 10.1016/j.tjnut.2025.101289, because it provides a nutrition-focused systematic review and meta-analysis on omega-3 fatty acids as a preventive strategy and is directly relevant to framing omega-3 exposure, prevention claims, and evidence limitations; 2. PMID: 40987202; doi: 10.1016/j.jnha.2025.100632, because it evaluates omega-3 intake in relation to diabetic microvascular outcomes and helps contextualize dietary PUFA intake as a systemic metabolic and inflammatory exposure; 3. PMID: 41935442; doi: 10.1016/j.redox.2025.103976, because mitochondrial dysfunction and oxidative stress are relevant to mechanistic discussion of diet, inflammation, sleep disruption, and carcinogenesis; 4. PMID: 41038126; doi: 10.1016/j.jdiacomp.2025.109178, because lipid-modifying and metabolic intervention evidence provides useful translational context for interpreting lipid-related exposures and disease outcomes without overstating causality.

The manuscript also requires final PLOS-specific compliance and language correction. The Data Availability statement says that all data are fully available and later states that datasets are available within the manuscript and supporting information files; the authors should ensure that the minimal anonymized dataset and analytic code are actually supplied in Supporting Information or a repository. The funding statement should include author initials, grant numbers, full funder names, funder URLs, and a clear statement on whether the funders had any role in study design, data collection, analysis, decision to publish, or manuscript preparation. The manuscript should also standardize PUFA, SQFFQ, OR, CI, BMI, Model 1, Model 2, passive smoking, occupational exposure, hot cooking oil exposure, healthy sleep score, and conditional logistic regression terminology. Minor grammar and formatting issues remain, including spacing before references and inconsistent use of “southwest China” versus “southwest, China.”

Overall, this revised manuscript addresses an important public-health question and presents potentially useful evidence regarding dietary PUFA intake, sleep patterns, and lung cancer odds in southwest China. However, the authors must further moderate causal language, clarify case and control ascertainment, strengthen dietary and sleep exposure reporting, present interaction analyses more cautiously, correct PLOS data and funding statements, add the recommended references, and polish language and formatting. I recommend minor revision. The manuscript can be accepted after minor revision if these issues are addressed carefully and consistently.

Reviewer #2: General Comments

The authors have adequately revised the manuscript in response to the previous comments. The revised version is clearer and better organized than the original submission. The rationale linking dietary PUFAs, sleep patterns, and lung cancer has been strengthened, the objectives are now more clearly stated, and the statistical methods have been described more appropriately. The interpretation of the stratified analyses has also been moderated, with appropriate acknowledgement that the interaction tests were not statistically significant.

Overall, the manuscript has improved and is now suitable for publication after addressing a few minor issues.

Minor Comments

1. The authors have acknowledged the limitation regarding smoking adjustment in the Discussion. However, considering the major role of smoking in lung cancer, this limitation should be emphasized slightly more. If detailed smoking information (e.g., pack-years) is available, its inclusion would strengthen the analysis. If not, this should be clearly stated as a limitation.

2. For omega-6 and total PUFAs, the continuous analyses were not statistically significant after full adjustment, whereas the tertile analyses remained significant. A brief explanation for this difference (such as possible non-linear associations or threshold effects) would improve the discussion.

3. The reported registration date appears to be later than the study period. The authors should clarify whether the study was registered retrospectively.

4. The overall language has improved. However, a final proofreading would help to correct a few minor grammatical errors and improve readability.

Final Recommendation

The major concerns raised in the previous review have been satisfactorily addressed. The remaining issues are minor and can be resolved through revision of the manuscript. I recommend Minor Revision.

**********

what does this mean?). If published, this will include your full peer review and any attached files.

If you choose “no”, your identity will remain anonymous but your review may still be made public.

**Do you want your identity to be public for this peer review?**  For information about this choice, including consent withdrawal, please see our Privacy Policy

Reviewer #1: No

Reviewer #2: No

**********

[NOTE: If reviewer comments were submitted as an attachment file, they will be attached to this email and accessible via the submission site. Please log into your account, locate the manuscript record, and check for the action link "View Attachments". If this link does not appear, there are no attachment files.]

To ensure your figures meet our technical requirements, please review our figure guidelines: https://journals.plos.org/plosone/s/figures

You may also use PLOS’s free figure tool, NAAS, to help you prepare publication quality figures: https://journals.plos.org/plosone/s/figures#loc-tools-for-figure-preparation.

NAAS will assess whether your figures meet our technical requirements by comparing each figure against our figure specifications.

Attachment Submitted filename: Reviewer report for revised version.pdf

# Author comment: Author response to Decision Letter 2

**DOI:** 10.1371/journal.pone.0358576.r004

16 Jul 2026

Dear reviewers and Editor,

We have revised our paper according to each of the comments, and revisions were highlighted in red to make sure changes could be easily visible to you and reviewers. We have uploaded 'Response to Reviewers' file to explain point-by-point the details of the revisions in the manuscript and our responses to the reviewers' comments.

Attachment Submitted filename: Response_to_reviewers_auresp_2.docx

# Peer review: Decision Letter 2

**DOI:** 10.1371/journal.pone.0358576.r005

**Authors:** Assoc. Prof. Phakkharawat Sittiprapaporn

14 Aug 2026

Dear Dr. Chen,

Thank you for submitting your manuscript to PLOS ONE. After careful consideration, we feel that it has merit but does not fully meet PLOS ONE’s publication criteria as it currently stands. Therefore, we invite you to submit a revised version of the manuscript that addresses the points raised during the review process.

Please submit your revised manuscript by Sep 28 2026 11:59PM. If you will need more time than this to complete your revisions, please reply to this message or contact the journal office at plosone@plos.org. When you're ready to submit your revision, log on to https://www.editorialmanager.com/pone/ and select the 'Submissions Needing Revision' folder to locate your manuscript file.

- A letter that responds to each point raised by the academic editor and reviewer(s). You should upload this letter as a separate file labeled 'Response to Reviewers'.
- A marked-up copy of your manuscript that highlights changes made to the original version. You should upload this as a separate file labeled 'Revised Manuscript with Track Changes'.
- An unmarked version of your revised paper without tracked changes. You should upload this as a separate file labeled 'Manuscript'.

If you would like to make changes to your financial disclosure, please include your updated statement in your cover letter. Guidelines for resubmitting your figure files are available below the reviewer comments at the end of this letter.

If applicable, we recommend that you deposit your laboratory protocols in protocols.io to enhance the reproducibility of your results. Protocols.io assigns your protocol its own identifier (DOI) so that it can be cited independently in the future. For instructions see: https://journals.plos.org/plosone/s/submission-guidelines#loc-laboratory-protocols. Additionally, PLOS ONE offers an option for publishing peer-reviewed Lab Protocol articles, which describe protocols hosted on protocols.io. Read more information on sharing protocols at https://plos.org/protocols?utm_medium=editorial-email&utm_source=authorletters&utm_campaign=protocols.

As the corresponding author, your ORCID iD is verified in the submission system and will appear in the published article. PLOS supports the use of ORCID, and we encourage all coauthors to register for an ORCID iD and use it as well. Please encourage your coauthors to verify their ORCID iD within the submission system before final acceptance, as unverified ORCID iDs will not appear in the published article. *Only*  the individual author can complete the verification step; PLOS staff *cannot*  verify ORCID iDs on behalf of authors.

We look forward to receiving your revised manuscript.

Kind regards,

Assoc. Prof. Phakkharawat Sittiprapaporn, Ph.D.

Academic Editor

PLOS One

**Journal Requirements:**

If the reviewer comments include a recommendation to cite specific previously published works, please review and evaluate these publications to determine whether they are relevant and should be cited. There is no requirement to cite these works unless the editor has indicated otherwise.

Please review your reference list to ensure that it is complete and correct. If you have cited papers that have been retracted, please include the rationale for doing so in the manuscript text, or remove these references and replace them with relevant current references. Any changes to the reference list should be mentioned in the rebuttal letter that accompanies your revised manuscript. If you need to cite a retracted article, indicate the article’s retracted status in the References list and also include a citation and full reference for the retraction notice.

**Additional Editor Comments:**

The authors have substantially improved the manuscript in this revision (R2), addressing the core methodological and structural concerns raised previously. However, a few minor issues remain that require brief clarification before final consideration, including

- Explicitly acknowledging the lack of cumulative smoking exposure data (pack-years) as a limitation.
- Expanding the discussion slightly regarding the non-linear/threshold dynamics observed in omega-6 and total PUFA tertile models vs. continuous models.
- Clarifying study registry status and retrospective timing, if applicable.
- Correcting a minor typesetting error in the abstract.

**Reviewers' comments:**

**Reviewer's Responses to Questions**

**Comments to the Author**

Reviewer #1: (No Response)

Reviewer #2: (No Response)

**********

2. Is the manuscript technically sound, and do the data support the conclusions?

Reviewer #1: (No Response)

Reviewer #2: Yes

**********

3. Has the statistical analysis been performed appropriately and rigorously?

Reviewer #1: (No Response)

Reviewer #2: Yes

**********

4. Have the authors made all data underlying the findings in their manuscript fully available?

The PLOS Data policy

Reviewer #1: (No Response)

Reviewer #2: Yes

**********

5. Is the manuscript presented in an intelligible fashion and written in standard English?

Reviewer #1: (No Response)

Reviewer #2: Yes

**********

Reviewer #1: Great work to be accepted.

Reviewer #2: The authors have made commendable improvements in this revised manuscript. The narrative, statistical presentation, and rationale have been significantly strengthened. The remaining issues are very minor and aim solely at ensuring methodological transparency, epidemiological clarity, and proper formatting before publication.

Comments to the Authors:

Smoking Confounding & Pack-Years (Limitation):

While adjusting for categorical smoking status (never/current/former) and passive smoking is appreciated, smoking remains the predominant risk factor for lung cancer. If cumulative smoking exposure (e.g., pack-years or daily intensity/duration) was not collected, please explicitly state this in the Limitations section as a potential source of residual confounding.

Discussion of Continuous vs. Tertile PUFA Findings:

In the results, continuous analyses for omega-6 and total PUFA intake were non-significant after full adjustment, whereas tertile-based analyses showed significant inverse associations. Please add a brief paragraph in the Discussion addressing this discrepancy (e.g., discussing potential non-linear relationships, threshold/plateau effects, or narrow intake ranges).

Study Registration:

Please clarify in the Methods section whether the study was registered in a clinical/observational study registry, and explicitly state if registration was retrospective relative to the study recruitment period (August 2023 – September 2024), including the registration ID and date.

**********

what does this mean?). If published, this will include your full peer review and any attached files.

If you choose “no”, your identity will remain anonymous but your review may still be made public.

**Do you want your identity to be public for this peer review?**  For information about this choice, including consent withdrawal, please see our Privacy Policy

Reviewer #1: No

Reviewer #2: No

**********

[NOTE: If reviewer comments were submitted as an attachment file, they will be attached to this email and accessible via the submission site. Please log into your account, locate the manuscript record, and check for the action link "View Attachments". If this link does not appear, there are no attachment files.]

To ensure your figures meet our technical requirements, please review our figure guidelines: https://journals.plos.org/plosone/s/figures

You may also use PLOS’s free figure tool, NAAS, to help you prepare publication quality figures: https://journals.plos.org/plosone/s/figures#loc-tools-for-figure-preparation.

NAAS will assess whether your figures meet our technical requirements by comparing each figure against our figure specifications.

# Author comment: Author response to Decision Letter 3

**DOI:** 10.1371/journal.pone.0358576.r006

23 Aug 2026

Dear Editors and Reviewers,

We have carefully revised our manuscript in response to all comments and suggestions. All changes in the revised manuscript have been highlighted in red for ease of review. We have also uploaded a separate “Response to Reviewers’ Comments” file, which provides detailed point-by-point responses to each comment and explains the corresponding revisions made in the manuscript.

We sincerely appreciate the valuable comments and suggestions from the Editors and Reviewers, which have helped us further improve the quality of our manuscript.

Attachment Submitted filename: Response_to_reviewers_auresp_3.docx

# Peer review: Decision Letter 3

**DOI:** 10.1371/journal.pone.0358576.r007

**Authors:** Assoc. Prof. Phakkharawat Sittiprapaporn

2 Sep 2026

Associations of dietary polyunsaturated fatty acids and sleep patterns with lung cancer in southwest China: a case-control study

PONE-D-26-10673R3

Dear Dr. Chen,

We’re pleased to inform you that your manuscript has been judged scientifically suitable for publication and will be formally accepted for publication once it meets all outstanding technical requirements.

Within one week, you’ll receive an e-mail detailing the required amendments. When these have been addressed, you’ll receive a formal acceptance letter and your manuscript will be scheduled for publication.

An invoice will be generated when your article is formally accepted. Please note, if your institution has a publishing partnership with PLOS and your article meets the relevant criteria, all or part of your publication costs will be covered. Please make sure your user information is up-to-date by logging into Editorial Manager at Editorial Manager® and clicking the ‘Update My Information' link at the top of the page. For questions related to billing, please contact billing support.

If your institution or institutions have a press office, please notify them about your upcoming paper to help maximize its impact. If they’ll be preparing press materials, please inform our press team as soon as possible -- no later than 48 hours after receiving the formal acceptance. Your manuscript will remain under strict press embargo until 2 pm Eastern Time on the date of publication. For more information, please contact onepress@plos.org.

Kind regards,

Assoc. Prof. Phakkharawat Sittiprapaporn, Ph.D.

Academic Editor

PLOS One

Additional Editor Comments (optional):

Reviewers' comments:

Reviewer's Responses to Questions

**Comments to the Author**

Reviewer #2: All comments have been addressed

**********

2. Is the manuscript technically sound, and do the data support the conclusions?

Reviewer #2: Yes

**********

3. Has the statistical analysis been performed appropriately and rigorously?

Reviewer #2: Yes

**********

4. Have the authors made all data underlying the findings in their manuscript fully available?

The PLOS Data policy

Reviewer #2: Yes

**********

5. Is the manuscript presented in an intelligible fashion and written in standard English?

Reviewer #2: Yes

**********

Reviewer #2: Comments to the Authors

The authors have thoroughly addressed all of my comments in Revision 3:

Smoking adjustment: The explicit limitation regarding the lack of quantitative pack-year data has been clearly incorporated into the Discussion.

Modeling discrepancy: The authors added a clear explanation in the Discussion addressing the potential non-linear or threshold dynamics explaining the difference between continuous and tertile models for omega-6 and total PUFAs.

Trial registration: Retrospective registration has been explicitly clarified in the Methods section.

The manuscript is now suitable for publication.

Recommendation: Accept

**********

what does this mean?). If published, this will include your full peer review and any attached files.

If you choose “no”, your identity will remain anonymous but your review may still be made public.

**Do you want your identity to be public for this peer review?**  For information about this choice, including consent withdrawal, please see our Privacy Policy

Reviewer #2: No

**********

# Editor report: Acceptance letter

**DOI:** 10.1371/journal.pone.0358576.r008

**Authors:** Assoc. Prof. Phakkharawat Sittiprapaporn

PONE-D-26-10673R3

PLOS One

Dear Dr. Chen,

I'm pleased to inform you that your manuscript has been deemed suitable for publication in PLOS One. Congratulations! Your manuscript is now being handed over to our production team.

At this stage, our production department will prepare your paper for publication. This includes ensuring the following:

* All references, tables, and figures are properly cited

* All relevant supporting information is included in the manuscript submission,

* There are no issues that prevent the paper from being properly typeset

You will receive further instructions from the production team, including instructions on how to review your proof when it is ready. Please keep in mind that we are working through a large volume of accepted articles, so please give us a few days to review your paper and let you know the next and final steps.

Lastly, if your institution or institutions have a press office, please let them know about your upcoming paper now to help maximize its impact. If they'll be preparing press materials, please inform our press team within the next 48 hours. Your manuscript will remain under strict press embargo until 2 pm Eastern Time on the date of publication. For more information, please contact onepress@plos.org.

You will receive an invoice from PLOS for your publication fee after your manuscript has reached the completed accept phase. If you receive an email requesting payment before acceptance or for any other service, this may be a phishing scheme. Learn how to identify phishing emails and protect your accounts at https://explore.plos.org/phishing.

If we can help with anything else, please email us at customercare@plos.org.

Thank you for submitting your work to PLOS One and supporting open access.

Kind regards,

PLOS One Editorial Office Staff

on behalf of

Assoc. Prof. Dr. Assoc. Prof. Phakkharawat Sittiprapaporn

Academic Editor

PLOS One
